# Engineering H_2_O_2_ Self-Supplying Platform for Xdynamic Therapies via Ru–Cu Peroxide Nanocarrier: Tumor Microenvironment-Mediated Synergistic Therapy

**DOI:** 10.1021/acsami.3c18888

**Published:** 2024-04-30

**Authors:** Worku Batu Dirersa, Tzu-Chun Kan, Jungshan Chang, Girum Getachew, Sonjid Ochirbat, Shamsa Kizhepat, Aswandi Wibrianto, Akash Rasal, Hung-An Chen, Anil Vithal Ghule, Tzung-Han Chou, Jia-Yaw Chang

**Affiliations:** † Department of Chemical Engineering, National Taiwan University of Science and Technology, Taipei 106335, Taiwan, Republic of China; ‡ Graduate Institute of Medical Sciences, College of Medicine, Taipei Medical University, Taipei 11031, Taiwan; § International Master/Ph.D. Program in Medicine, College of Medicine, Taipei Medical University, Taipei 11031, Taiwan; ∥ International Ph.D. Program for Cell Therapy and Regeneration Medicine, College of Medicine, Taipei Medical University, Taipei 11031, Taiwan; ⊥ Green Nanotechnology Laboratory, Department of Chemistry, Shivaji University, Kolhapur 416004, India; # Department of Chemical and Materials Engineering, National Yunlin University of Science and Technology, Yunlin 64002, Taiwan, Republic of China

**Keywords:** reactive oxygen species, camptothecin, Fenton reactions, oxygen vacancy, glutathione depletion, synergistic therapy

## Abstract

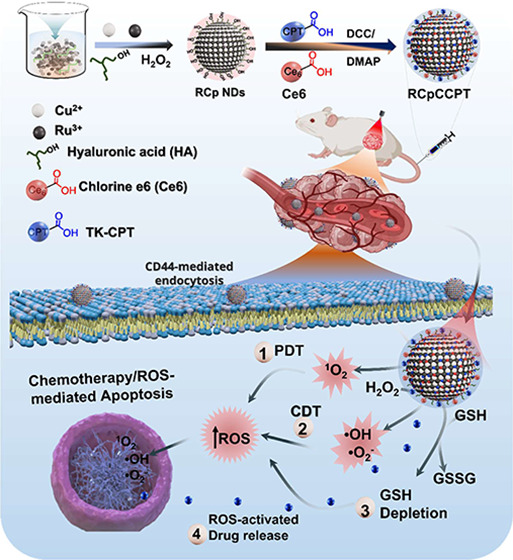

Of the most common, hypoxia, overexpressed glutathione (GSH), and insufficient H_2_O_2_ concentration in the tumor microenvironment (TME) are the main barriers to the advancment of reactive oxygen species (ROS) mediated Xdynamic therapies (X = photo, chemodynamic, chemo). Maximizing Fenton catalytic efficiency is crucial in chemodynamic therapy (CDT), yet endogenous H_2_O_2_ levels are not sufficient to attain better anticancer efficacy. Specifically, there is a need to amplify Fenton reactivity within tumors, leveraging the unique attributes of the TME. Herein, for the first time, we design Ru_*x*_Cu_1–*x*_O_2_–Ce6/CPT (RCpCCPT) anticancer nanoagent for TME-mediated synergistic therapy based on heterogeneous Ru–Cu peroxide nanodots (Ru_*x*_Cu_1–*x*_O_2_ NDs) and chlorine e6 (Ce6), loaded with ROS-responsive thioketal (TK) linked-camptothecin (CPT). The Ru–Cu peroxide NDs (RCp NDs, *x* = 0.50) possess the highest oxygen vacancy (O_V_) density, which grants them the potential to form massive Lewis’s acid sites for peroxide adsorption, while the dispersibility and targetability of the NDs were improved via surface modification using hyaluronic acid (HA). In TME, RCpCCPT degrades, releasing H_2_O_2_, Ru^2+/3+^, and Cu^+/2+^ ions, which cooperatively facilitate hydroxyl radical (•OH) formation and deactivate antioxidant GSH enzymes through a cocatalytic loop, resulting in excellent tumor therapeutic efficacy. Furthermore, when combined with laser treatment, RCpCCPT produces singlet oxygen (^1^O_2_) for PDT, which induces cell apoptosis at tumor sites. Following ROS generation, the TK linkage is disrupted, releasing up to 92% of the CPT within 48 h. In vitro investigations showed that laser-treated RCpCCPT caused 81.5% cell death from PDT/CDT and chemotherapy (CT). RCpCCPT in cancer cells produces red-blue emission in images of cells taking them in, which allows for fluorescence image-guided Xdynamic treatment. The overall results show that RCp NDs and RCpCCPT are more biocompatible and have excellent Xdynamic therapeutic effectiveness in vitro and in vivo.

## 1 Introduction

ROS, which consists primarily of ^1^O_2_, •OH, and superoxide anions (^•^O_2_^–^) serve as significant regulatory and signaling agents within cells.^1,2^ It has been demonstrated that significant ROS buildup in tumor areas can cause increased oxidative stress in tumor cells, upsetting their redox balance and ultimately resulting in necrosis or apoptosis.^3,4^ Consequently, ROS-mediated treatments like CDT and PDT have gained widespread adoption in noninvasive tumor management due to their remarkable specificity and minimal susceptibility to drug resistance.^5,6^ Nevertheless, the insufficient amount of essential substrates within the TME, such as molecular oxygen (O_2_) for PDT and H_2_O_2_ for CDT, caused a significant challenge in ROS-based therapy.^7,2,8^ One example of a strategy employed to effectively generate O_2_ in a specific location is the utilization of catalase or catalase-like agents. This approach aims to mitigate hypoxia during PDT.^9,10^ In a similar vein, several substances, including B-apache, glucose oxidase, cisplatin, and CaO_2_, have been investigated for their potential to increase H_2_O_2_ concentrations, hence amplifying the effectiveness of CDT.^11^ Currently, many studies are dedicated to elevating H_2_O_2_ levels within tumors. Direct transport of H_2_O_2_ and glucose oxidase (GOx) to catalyze in situ H_2_O_2_ synthesis is one of the promising approaches in recent times.^12,13^ Nevertheless, challenges persist, primarily related to the unavoidable leakage of H_2_O_2_ throughout the movement of the bloodstream and the restricted catalytic effectiveness of GOx enzymes, particularly in hypoxic tumor circumstances.^14^ An alternative approach involves the utilization of CAT, ascorbic acid,^15^ or MnO_2_ as catalysts to facilitate the conversion of intracellular H_2_O_2_ into O_2_, hence mitigating the occurrence of hypoxia. Nevertheless, the supply of O_2_ derived from the breakdown of MnO_2_ and catalysis by CAT is similarly subject to depletion as a result of the restricted amounts of intratumoral H_2_O_2_.

Indeed, the potential applications of current PDT agents are constrained by the inherent oxygen depletion within hypoxic solid tumors. In these conditions, photosensitizers struggle to effectively generate ^1^O_2_ due to a lack of sufficient oxygen supply within the tumors.^16−18^ This insufficiency in oxygen sources results in reduced production of ROS significantly diminishing the effectiveness of treatments reliant on O_2_, such as PDT and radiation therapy (RT).^19−21^ To composite this issue, PDT agents rapidly deplete local oxygen, exacerbating tumor hypoxia, which in turn leads to even lower and unsustainable PDT effectiveness.^22−24^ Furthermore, certain drugs also struggle to designate effectiveness within a hypoxic TME. Consequently, it becomes imperative to adjust the hypoxic TME to enhance the effectiveness of antitumor treatments. To tackle the issue of insufficient oxygenation in tumor hypoxia, numerous approaches have been suggested to enhance oxygen levels inside the hypoxic TME. The aforementioned strategies encompass the repair of tumor vasculature and the provision of in situ oxygen self-supply.^25,26^ In the quest to generate oxygen in situ, researchers have explored the potential of leveraging endogenous H_2_O_2_, which is more abundant in cancer cells compared to healthy cells.^27^ Even though tumor cells have H_2_O_2_ concentrations five times higher than normal cells, the intracellular H_2_O_2_ levels are still insufficient to achieve effective CDT.^28,29^ As a result, there is a need to develop novel approaches and materials to modulate and enhance local or intratumoral oxygen levels. This is crucial for overcoming the primary limitation of most current photosensitizers, enhancing PDT, and enhancing the efficacy of CDT in the therapy of solid tumors.

Fortunately, metal peroxides (MO_2_) have been intensively studied for their ability to address tumor hypoxia via a disproportionation reaction with H_2_O_2_ in tumor tissue. In this context, the capacity of MO_2_ to generate H_2_O_2_ introduces the potential for developing cascade Fenton nanoagents for catalytic nanotherapeutics. MO_2_ generally comprises metal ions and peroxo groups, which can undergo a reaction with H_2_O resulting in the formation of H_2_O_2_.^30−32^ The H_2_O_2_ produced in this process has various valuable applications in the field of biomedicine.^33,34^ This self-generated H_2_O_2_ can serve as a reactant in Fenton (like) catalytic reactions, leading to the production of a large number of •OH for cancer nanomedicine.^33,35,36^ Additionally, H_2_O_2_ can self-decay to generate O_2_, enhancing the therapeutic effectiveness of different O_2_-dependent modalities such as PDT and RT.^37,38^

**Scheme 1:**
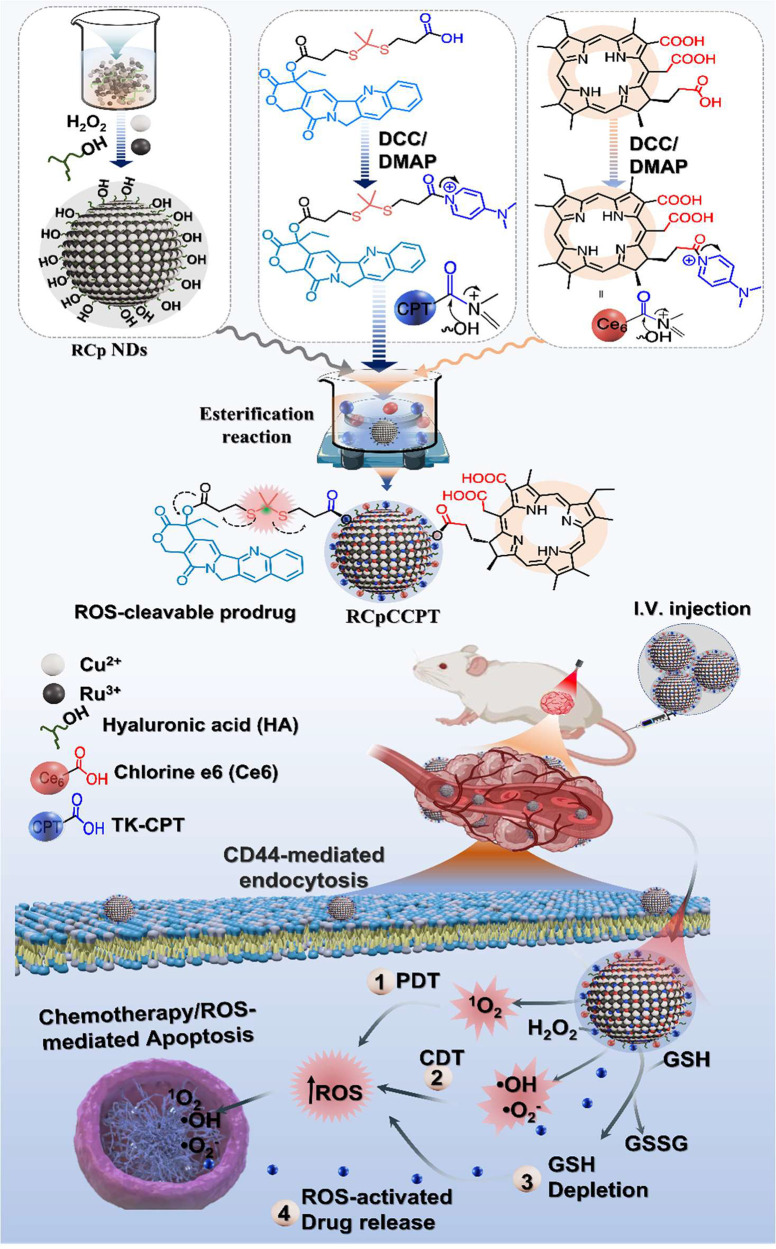
Schematic Illustration of the Synthesis and Intracellular Synergetic Catalytic Loop of RCpCCPT in TME Promoted PDT in the Presence of Light Source, H_2_O_2_-Self-Supplied for CDT, GSH Depletion, and ROS-Induced Chemotherapeutic Activity, and the Mechanism Nanodots Interlinked with the TK-CPT and Ce6 through the Formation of Ester Bond and Xdynamic Therapeutic Illustration

In this study, we aim to address the challenges associated with limited H_2_O_2_ availability in the TME while recognizing the potential of MO_2_. To overcome these limitations and leverage the advantages of MO_2_, we have designed a multitherapeutic platform named RCpCCPT, utilizing the Ru–Cu cocatalytic properties and a nonstoichiometric ratio of mixed valence state ion pairs as core facilitators. The standard reduction potentials of the Ru^3+^/Ru^0^ couple versus the standard hydrogen electrode (0.68 V versus SHE) are significantly higher than those of the Cu^2+^/Cu^0^ couple (0.34 V versus SHE).^39,40^ This implies that Ru ions can effectively displace Cu ions, and the corresponding chemical equation can be represented as Cu + Ru^3+^ → Cu^2+^ + Ru. As a result, the novel RCp NDs comprise a mixed valence ratio of the metal ion pair and stimulate the formation of oxygen vacancies on the surface of nanodots, which act as peroxo (O–O) acceptor.^41,42^ Thus, it aimed to improve cancer therapy by utilizing a nanocarrier composed of Ru–Cu-peroxide, which accelerates the Fenton reaction in a TME-responsive manner, thereby boosting the effectiveness of CDT. Simultaneously, we explore the influence of RCpCCPT on PDT, and CT by incorporating Ce6 as the photosensitizer and CPT as the anticancer agent. The RCpCCPT exhibits tumor cell targeting capabilities through CD44-mediated endocytosis trafficking, leveraged by the HA effect. The collaborative interaction between Ru^2+/3+^ and Cu^+/2+^ redox pair ions effectively enhances the Fenton reaction via the Ru–Cu catalytic loop, resulting in elevated quantities of lethal •OH within tumors, in comparison to the presence of individual ion pairs. Moreover, the remarkable H_2_O_2_ generation capability of RCpCCPT helps to the mitigation of tumor hypoxia which has been a significant challenge in clinical solid tumor treatment. The additional H_2_O_2_ ensures the effectiveness of Ru^3+^/Cu^2+^-mediated CDT/PDT and also depletes overexpressed GSH, boosting TK linker bond breakage and initiating CT by generating a massive amount of ROS in the TME. The collaborative effects of CDT/PDT features in RCpCCPT allow for precise multitherapeutic action through programmed and efficient drug release (Scheme 1).

## 2 Experimental Section

The experimental section provides an in-depth discussion of several procedure sections, encompassing the description of the chemicals and reagents utilized, the establishment of a standardized investigation procedure, the intracellular detection of GSH, and the utilization of specific instruments for material characterizations, as detailed in the Supporting Information.

### 2.1 Synthesis of Ru_*x*_Cu_1–*x*_O_2_ NDs

HA-wrapped Ru_*x*_Cu_1–*x*_O_2_ NDs were synthesized using a wet chemical approach conducted at ambient conditions. Simply, HA (80 mg), 10 mL of 8.5 mg/mL CuCl_2_.2H_2_O solution, and Ru precursor (x = 0.0, 0.25, 0.50, 0.75, and 1.00) out of 5.25 mg/mL RuCl_3_.2H_2_O were added to the solution, along with 0.2 M NaOH (25 μL) and 30% H_2_O_2_ (400 μL). The solution was agitated for 30 min. The resulting HA-coated Ru_*x*_Cu_1–*x*_O_2_ NDs were collected using ultrafiltration and washed multiple times with a mixture of water and alcohol. The Ru–Cu oxide was prepared without adding H_2_O_2_ as a comparison.

### 2.2 Colorimetric Assay of Peroxo Groups and •OH Formation

To evaluate the formation of peroxo groups, we first dissolved KMnO_4_ (0.1 M) in an acidic solution (0.1 M, H_2_SO_4_). This solution was then exposed to RCp NDs. As a reference, a solution with the same concentration of KMnO_4_ was treated with 50 mM H_2_O_2_. Afterward, the absorption spectra were measured within the wavelength range of 400 to 650 nm.

The peroxidase-like activity of RCp NDs was demonstrated by varying pH values, H_2_O_2_, and RCp NDs concentrations. The kinetics of •OH formation was also investigated by altering the concentrations of RCp NDs. Formation of •OH detected using TMB probe at λ_max._ = 652 nm absorbance. Control groups were established using TMB solutions treated with either RCP NDs or H_2_O_2_ alone. In the experimental procedure, a mixture was prepared consisting of 50 μL of TMB solution (10 mM in DMSO), 75 μL of H_2_O_2_ (50 mM), and 250 μL of PBS (pH = 5.5) in a quartz cuvette. Subsequently, 50 μL of RCp NDs (100 ppm) were introduced into the mixture, and the formation of oxidized TMB (oxTMB) was quantified at λ_max._ = 652 nm. This process was repeated with variations in pH value, H_2_O_2_ concentration, and RCp NDs concentration.^43^

### 2.3 Extracellular GSH Depletion

The ability of RCp NDs to neutralize the GSH substrate was assessed using Ellman’s assay.^44,45^ GSH contains thiol groups that can interact with Ru^2+/3+^ and Cu^+/2+^ ions, leading to the formation of GSSG intermediary substances that exhibit an absorbance peak at around 412 nm. In the experiment, 250 μL of RCp NDs were combined with 200 μL of PBS and 50 μL of GSH (1 mM, pH = 5.0). Afterward, 50 mL of this solution was mixed with 0.2 μL of DTNB (50 mM in DMSO), and the absorption spectrum was measured every 10 min using UV–vis absorption spectroscopy. The effects of this interaction were studied over time and varied based on the concentration of RCp NDs.

### 2.4 O_2_ Generation Activity

The capacity of Ru_*x*_Cu_1–*x*_O_2_ NDs (x = 0.0, 0.25, 0.50, 0.75, 1.00) to produce O_2_ was evaluated by mixing them in a pH buffer solution (pH 5.5) at equal concentrations (100 ppm), and compared with their precursors. The concentration of dissolved oxygen in the solution was monitored at 5 min intervals using a dissolved oxygen meter (Lutron DO-5509). To simulate intracellular O_2_ generation, the experiment was conducted by exposing cancer cells to RCp NDs as an O_2_ supplier, and Ru(dpp)_3_Cl_2_ as an oxygen sensor, then followed by assessment of intracellular O_2_ levels using Ru(dpp)_3_Cl_2_, which interacts with O_2_ formed due to peroxide dissociation in the system. Lastly, confocal imaging was employed to detect the fluorescence of Ru(dpp)_3_Cl_2_, and the results were compared with the control.

### 2.5 Fabrication of ROS-Responsive RCpCCPT

The synthesis of RCpCCPT involved linking the carboxylic group of TK linker-CPT and Ce6 in a 1:1 proportion to RCp NDs through an esterification procedure. This reaction was facilitated using dicyclohexylcarbodiimide as a coupling agent and 4-(dimethylamino)-pyridine as a catalyst, known as DCC/DMAP coupling chemistry. Initially, the carboxylic group of the TK linker-CPT was stimulated through the reaction of 5 mg of CPT-TK with DCC/DMAP (1:1) in 5 mL DMSO. Similarly, the carboxylic group of the Ce6 (5 mg) was activated by adding DCC/DMAP (1:1) in 5 mL DMSO in another vial. Subsequently, both solutions were agitated in the absence of light at room temperature for 30 min. Then, both solutions were introduced concurrently into a 20 mL solution of RCp NDs at a concentration of 500 ppm. The mixture was then gently stirred at room temperature for a duration of 24 h. Afterward, the solution underwent centrifugation at a speed of 6000 rpm for 10 min. The resulting liquid above the sediment was then subjected to dialysis using a 1 KDA (MWCO) membrane to eliminate any remaining unreacted CPT and Ce6 derivatives. Subsequently, the liquid portion of RCpCCPT was subjected to freeze-drying. The predetermined mass of RCpCCPT was employed to ascertain the content of the drug and the efficiency of encapsulation using UV–vis absorption spectroscopy at a wavelength of 366 nm. This was accomplished by correlating the data with the calibration curve (R^2^= 0.995).
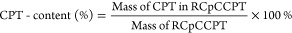

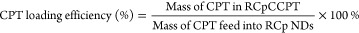


The experimental investigation involved conducting drug release studies of CPT at varying pH levels and under laser stimulation circumstances, utilizing a 100 mL volume of PBS. A 5 mL solution of RCpCCPT with a concentration of 100 ppm was contained within a dialysis bag. The bag was then submerged in a solution of PBS and subjected to dialysis for a duration of 48 h. The dialysis process was carried out under controlled conditions, specifically at a fixed rotational speed of 400 rpm at a temperature of 37 °C. The dialysis procedure was conducted using varying pH levels, specifically 5.5, 6.5, and 7.4, to examine the impact of pH on the release of the drug. The aforementioned procedure was repeated under the conditions of a laser with a wavelength of 671 nm and an intensity of 1.0 W/cm^2^ in the presence of H_2_O_2_ at different pH levels. At specified time intervals, a volume of 1 mL of the sample solution was extracted from the dialysis medium. This was then substituted with an equal volume of fresh PBS to ensure that the total volume of the external phase remained constant. The absorbance at a wavelength of 366 nm was subsequently evaluated using UV–vis absorption spectroscopy. The estimation of CPT concentration at each time interval was conducted about the calibration curve, which exhibited a coefficient of determination R^2^ = 0.995.

### 2.6 Evaluation of ROS-Generations

Two different methods were conducted to examine the ROS generation capacity of RCpCCPT. Particularly, 1,3-diphenylisobenzofuran (DPBF) and TMB probes are used for ^1^O_2_ and •OH radical detection, respectively. Briefly, DPBF was combined with RCpCCPT in DMSO and then transferred into a quartz cuvette. Next, the solution was subjected to laser radiation with a wavelength of 671 nm, at a power density of 1.0 W/cm^2^, for 5 min intervals. This process was continued for a total duration of 45 min. The UV–vis absorption spectroscopy was employed to analyze the absorption spectra of DPBF at a specific wavelength of 416 nm. This was done to examine any alterations associated with the generation of ^1^O_2_. Additionally, a control experiment was performed to compare the results by utilizing DPBF while subjecting it to laser irradiation.

Furthermore, the electron spin resonance (ESR) technique was employed to investigate the specific type of ROS produced by RCpCCPT. The experiment employed the radical trappers 100 mM TEMP (in DMSO) and 100 mM DMPO (in DMSO and H_2_O) to identify the ^1^O_2_, ^•^O_2_^–^, and •OH entities. For this test, a total of 0.9 mL of RCpCCPT solution was mixed with 0.1 mL of either TEMP or DMPO spin-trapping agents. ESR spectra were recorded after 5 min of exposure to a 671 nm laser with an intensity of 1.0 W/cm^2^.

### 2.7 Invitro Cellular Investigations

#### 2.7.1 Biocompatibility of RCp NDs and RCpCCPT

In this work, human breast cancer cell lines, specifically MDA-MB-231 and 4T1, were utilized to evaluate both cytocompatibility and therapeutic efficacy. These cells were cultured under carefully controlled conditions; a 5% CO_2_ environment and a temperature of 37 °C. Specifically: For MDA-MB-231 cells, DMEM growth medium was used, supplemented with 10% fetal bovine serum, 1% nonessential amino acids, 1% antibiotic-antimycotic solution, 1% sodium pyruvate, and 1% l-glutamine. The 4T1 cells were cultured in RPMI-1640 media supplemented with 4500 mg/L of glucose, 1500 mg/L of sodium bicarbonate, and 10% fetal bovine serum. Additionally, the solution comprises 2 mM l-glutamine, 10 mM HEPES, and 1 mM each of sodium pyruvate. Using WST-1 assays, the cytotoxicity of RCp NDs was assessed. The 4T1 or MDA-MB-231 cells were initially placed in 96-well plates containing 100 μL of growth media. The cells were seeded at a density of roughly 1.0 × 10^5^ cells per well. Subsequently, to promote cellular adhesion, the plates underwent incubation for a duration of 24 h at a temperature of 37 °C within a humid environment containing 5% CO_2_. Following the initial incubation, the media was removed and the cells were subjected to a wash using PBS. Subsequently, the wells were replenished with fresh culture media supplemented with different concentrations of RCpCCPT solution. The cells were subsequently incubated at a temperature of 37 °C for an additional duration of either 24 or 48 h. Subsequently, a new aliquot of the medium was dispensed into each well, accompanied by the addition of 0.01 mL of the WST-1 reagent. Subsequently, the plate was subjected to an additional 30 min incubation period. The quantification of live cells was performed by measuring the absorbance at 450 nm using a microplate reader, namely the Bio-Tek Rad model. This method made it possible to evaluate the cytotoxicity of RCp NDs and determine their therapeutic effects on certain cancer cells.

#### 2.7.2 In Vitro Hemolysis Analysis

In vitro, hemolysis experiments were conducted as previously mentioned.^46,47^ In a nutshell, fresh whole blood samples from a healthy mouse were collected and stabilized using EDTA. The serum was separated from the entire blood using centrifugation at a speed of 3000 rpm for 10 min, to isolate the red blood cells (RBCs). Consequently, the RBCs underwent a cleaning process utilizing a triton solution, followed by dilution by the combination of 0.4 mL of RBCs with 1.6 mL of triton. A total volume of 50 μL of the diluted RBC solution was mixed with 50 μL of Triton, serving as a positive control. Additionally, 50 μL of PBS was used as a negative control. Furthermore, 50 μL of PBS buffer was individually combined with varying quantities of RCp NDs and RCpCCPT. The liquid portions were centrifuged at a rate of 3000 rpm for a period of 1 h. Subsequently, the absorbance spectra of 100 μL of the supernatants were recorded using a microplate spectrophotometer (BioTek, iMark). The estimation of hemolysis percentages in the samples was conducted utilizing the following equation:
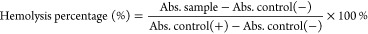
where Abs. represents the absorbance of the supernatants at a wavelength of 576 nm, as determined using a BioTek iMark microplate spectrophotometer.

#### 2.7.3 Cellular Uptaking and Endocytosis Pathway Studies

To validate the intracellular uptake of RCpCCPT by cancer cells, MDA-MB-231 cells were cultured at a density of 1 × 10^5^ cells per well overnight on a 6-well plate. This was done to ascertain the intracellular uptake of RCpCCPT by cancer cells. On the subsequent day, following a PBS rinse, the cancer cells were subjected to a treatment involving the addition of 0.1 mL of media containing RCpCCPT. Following a 24 h incubation period, the cells were subjected to an additional wash with PBS before a 10 min exposure to 2 mL of 70% alcohol. The cellular nucleus structure was monitored by utilizing a volume of 2 mL of NucGreen solutions.

In a six-well plate, MDA-MB-231 cancer cells were cultured for a duration of 24 h. The cells were preincubated using a culture mixture that consisted of several inhibitors, namely Me-CD (16 mM), genistein (100 g/mL), nystatin (50 g/mL), and amiloride hydrochloride (2.5 mM). Following the preincubation period, the medium was removed and substituted with RCpCCPT. Subsequently, the cells were subjected to an additional incubation period of 6 h. The experiment employed NucGreen as a staining agent for the cell nuclei. The cellular uptake of RCpCCPT was subsequently examined using CLSM to validate the process of endocytosis.

#### 2.7.4 Intracellular ROS-Assay

DCFH-DA was applied to investigate the formation of ROS within cells. MDA-MB-231 cells were grown overnight on 6-well cell chamber slides overnight. After removing the old medium, a fresh medium containing RCp NDs or RCpCCPT was introduced. After 6 h of incubation, DCFH-DA (25 μM) was added to demonstrate the production of ^1^O_2_ and placed for 20 min. Then, the cells were either left in the dark (control) or subjected to 671 nm laser light (PDT) at 1.0 W/cm^2^ for 5 min. They were first cleaned with PBS and then treated with 2 mL of 70% alcohol for 20 min before being washed once more with PBS. Finally, the cells were stained with DAPI (2 ppm), and CLSM was used to record fluorescence images.

#### 2.7.5 Cell Apoptosis and In Vitro Photo/Chemodynamic Efficacy

The MDA-MB-231 cell line was cultured in 6-well plates with a cell density of 1 × 10^6^ cells per well and incubated for a duration of 24 h. The cells were then put through several procedures, including the control (saline/H_2_O_2_), RCp NDs, RCpCCPT, and RCpCCPT/Laser. The experimental groups are all treated with 100 ppm of the corresponding material. After the treatment, the cells were cultivated for 24 h to assess the apoptosis level. Following incubation, 2 mL of trypsin was used to detach the cells, and then the cells were cleaned using PBS. Following a PBS wash, the cells were centrifuged to remove the PBS. 0.5 mL of binding buffer, 5 μL of PI, and 5 μL of FITC-annexin were added to interact with the cells. The cell solution was incubated for 30 min on ice to aid in the internalization of the probe. In the end, flow cytometry analysis was performed for apoptosis detection using the costaining agents, Annexin V-FITC/PI method.

### 2.8 Breast Cancer-Bearing Xenograft Model

The approach employed for xenograft models of breast cancer was derived from previously published studies.^48−50^ The study utilized female SCID mice, aged 5 weeks, obtained from BioLasco Company in Taipei, Taiwan. The mice were then housed at the animal facility of Taipei Medical University, where they were provided with unrestricted access to food and subjected to a 12-h light-dark cycle. After 1 week of adaptation, every mouse was implanted with 1 × 10^6^ MDA-MB-231 cells. The cells were mixed in equal proportions of PBS and Matrigel Matrix and then injected subcutaneously on the right flank of each mouse. Subsequently, the mice were divided into four distinct groups: the saline (I), RCp NDs (II), RCpCCPT (III), and RCpCCPT/Laser IV) groups. This division was done randomly once the tumor diameters reached around 50 mm^3^. Each group was administered a dual injection of the corresponding substances. The injection was delivered intravenously through the tail vein at a dosage of 5 mg/kg. The tumor size was measured using an electronic caliper to monitor its growth. The volume was calculated using the formula: width^2^ × length ×0.52. Body weight and tumor size were measured at 3 days per interval. The mice were euthanized on 21 days of their treatment to analyze their major organs, and tumor weight, and perform a biocompatibility test.

#### 2.8.1 In Vivo Biosafety Analysis

Biosafety analysis was performed on the MDA-MB-231 tumor-bearing mice following their in vivo treatment. Twenty-1 days after the development of tumors, mice were euthanized. Following collection, main organs and tumors were immersed in formaldehyde. Following organ harvest and formalin solution immersion, the specimens were processed for H&E staining to obtain a comprehensive visualization of the tissue morphology. To facilitate hematological and biochemical analyses, tubes containing EDTA were utilized to collect the whole blood from the sacrificed mice. In preparation for biochemical analysis, whole blood was obtained by centrifuging the blood cells for 5 min at 2000 rpm. The serum was then chilled to −20°c in a refrigerator in preparation for further analysis.

## 3 Results and Discussion

### 3.1 Preparation and Characterization of Ru_*x*_Cu_1–*x*_O_2_ NDs

**Figure 1:**
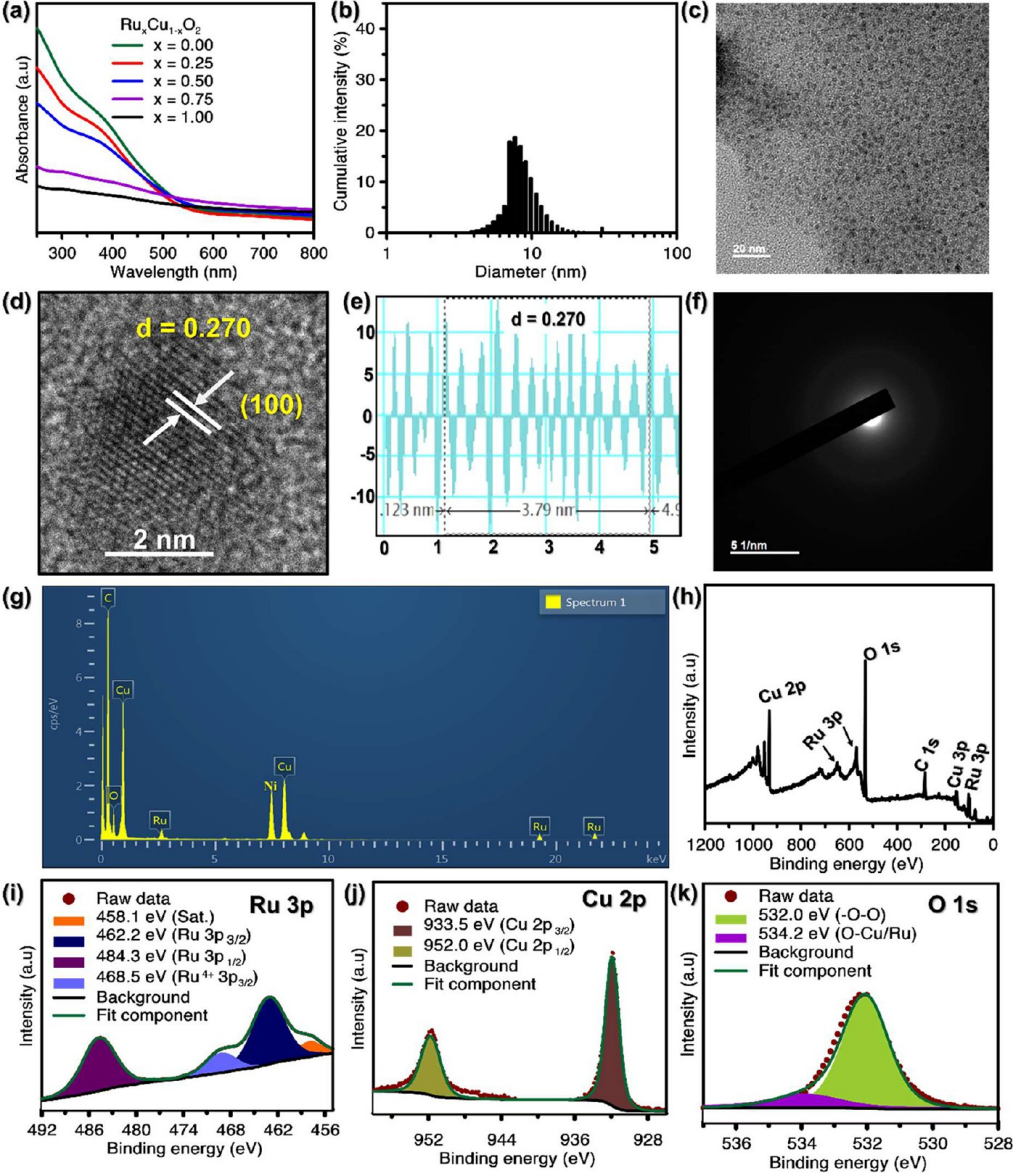
RCp NDs characterizations: (a) UV–visible absorption spectrum of Ru_*x*_Cu_1–*x*_O_2_ NDs (*x* = 0.0, 0.25, 0.50, 0.75), (b) DLS measurement, (c) HRTEM image, (d) *d*-spacing information, (e) *d*-spacing pattern, (f) SAED image, and (g) EDS spectrum. (h) XPS survey spectra of RCp NDs and their corresponding high-resolution spectra for (i) Ru 3p; (j) Cu 2p; (k) O 1s.

Bimetallic Ru_*x*_Cu_1–*x*_O_2_ NDs, were synthesized with various Ru to Cu ratios (x = 0.0, 0.25, 0.50, 0.75, and 1.00) to investigate their composition-dependent synergetic Fenton reaction and their ability to deplete overexpressed GSH in the TME, as depicted in Figure 1a. Afterward, these NDs were coated with HA through a straightforward wet chemical method, and their optical spectroscopic properties in an aqueous solution were analyzed using UV–vis absorption spectroscopy. The results showed that the RCp NDs exhibited a weak absorption band around 350 nm compared to Cu peroxide alone, and further the absorption decreased as the Ru concentration increased (Figure 1a). Moreover, they displayed a long tail absorption band extending up to 800 nm, which confirmed the successful incorporation of Ru and Cu in the RCp NDs. The results obtained from dynamic light scattering (DLS) studies (Figure 1b) demonstrate that the RCp NDs manufactured in this study were evenly distributed in water, exhibiting an average hydrodynamic diameter of 7.5 nm. This characteristic is particularly beneficial for passive cancer targeting. Additionally, TEM and high-resolution TEM (HRTEM) images demonstrated monodispersed RCp NDs with a size reached up to 4 nm (Figures S3 and 1c). The interplanar distance of RCp NDs was determined from the HRTEM picture, showing a highly crystalline nature with a lattice spacing of 0.270 nm (Figures 1d and 1e), corresponding to (100) lattice planes (Figure 1f). EDS spectra confirmed the presence of several compositional ingredients in RCp NDs, including Ru, Cu, and O, as illustrated (Figure 1g). The HRTEM elemental pictures (Figure S4) provide unambiguous visualization and verification of the distribution and composition of the key constituents of RCp NDs.

Furthermore, XPS was performed to investigate the elemental composition and chemical valence state of the elements in RCp NDs. The survey XPS spectra showed characteristic peaks at 100.01 and 150.0 eV, corresponding to Ru (3p) and Cu (3p), respectively (Figure 1h). Other at 300 and 530 eV refer to C 1s and O 1s, while the peaks around 600–700 eV and at 950.5 eV represent Ru 3p couple and Cu 2p, respectively, confirming that the RCp NDs contain principal precursors. In brief, magnified XPS data demonstrated that Ru exists in multiple chemical states such as Ru 3p_1/2_ at 484.5 eV, Ru 3p_3/2_ at 468.2 eV, and Ru^4+^ 3p_3/2_ at 462.2 eV, while Ru^2+/3+^ satellite peak observed at 458.1 eV, which is consistent with recent reports^51^ (Figure 1i). Similarly, Cu-derivatives; Cu 2p_1/2_ and Cu 2p_3/2_ were detected at 952.1 eV, and 933.5 eV respectively (Figure 1j). In the XPS analysis of RCp NDs, as shown in Figure 1k, two distinct peaks were seen in the O 1s region. The peak at 534.7 eV was attributed to the Ru/Cu–O bonding, while the peak at 532.6 eV was assigned to the O–O bonding, indicating the presence of peroxo groups. The XRD pattern of RCp NDs exhibited a crystal structure with reduced dimensions, indicating the presence of small grain sizes in the NDs. The prominent peaks at angles of 33.2° and 38.4° correspond to the distinctive signals emitted by the (100) and (101) lattice planes, respectively. These signals may be identified using the PDF numbers 870726 and 892531, as shown in Figure S 4d.

Surface functional groups on the RCp NDs were examined through FTIR analysis, revealing peaks at 3400 cm^–1^, which distinguish the O–H group in HA, as well as stretching vibrations of O–H and C–O at 1680 cm^–1^ (Figure S5). Zeta potentials of the as-synthesized RCp NDs were measured, and they were found to be strongly negative due to the charge-neutralizing HA on the NDs’ surface (Figure S 4f). This further suggested the good biocompatibility of RCp NDs (Figure S6).

### 3.2 H_2_O_2_-Self Producing and •OH Generation by RCp NDs

**Figure 2:**
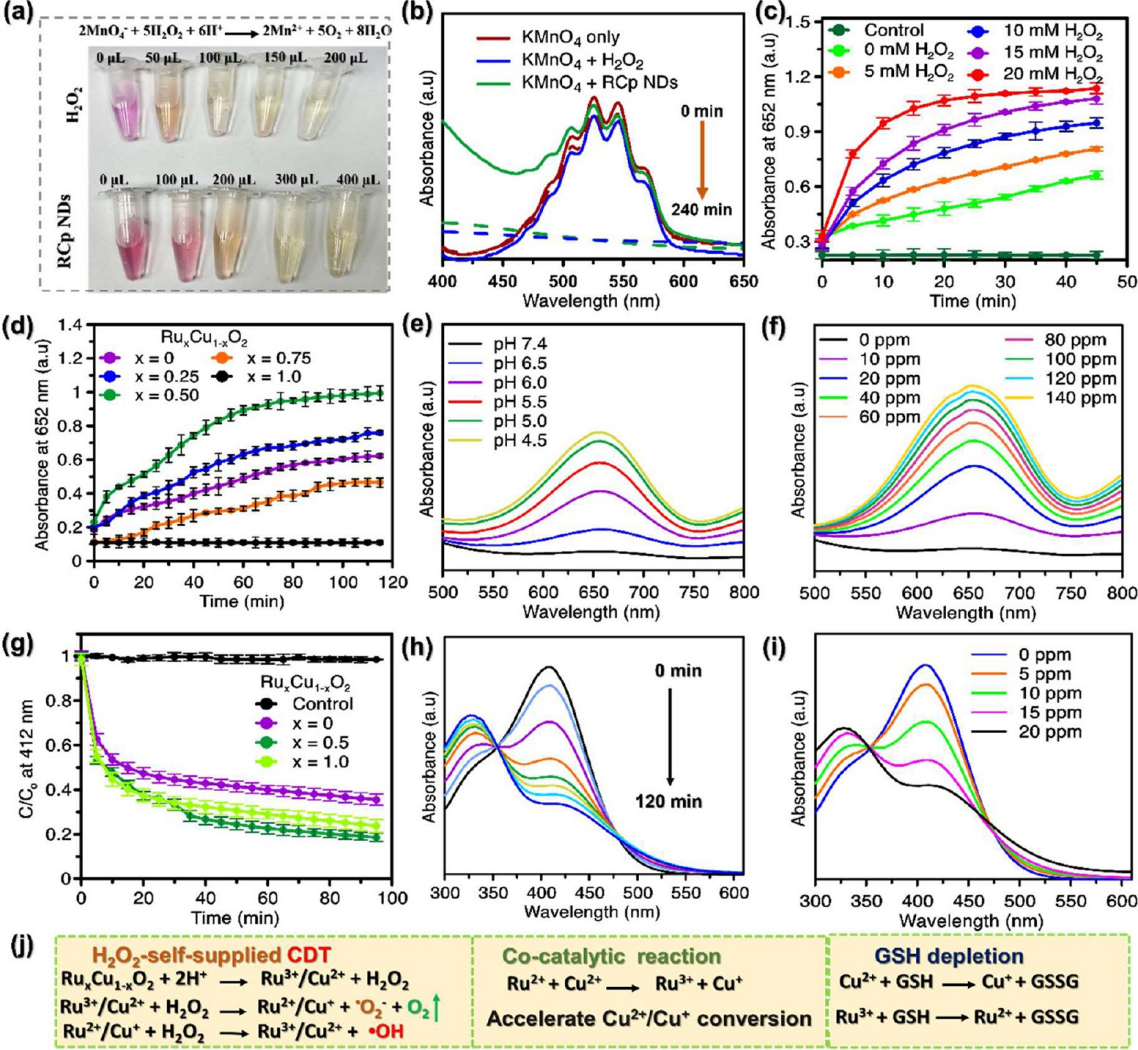
Fenton (like) reaction and GSH-depletion property of RCp NDs: (a) Colorimetric assay confirmation of peroxo-group in RCp NDs (50 ppm) using 0.1 mM KMnO_4_ probe solution; reduction of purple MnO_4_^–^ to colorless Mn^2+^ by peroxo-group in the acidic media, while 50 mM H_2_O_2_ used as reference. (b) UV–vis absorption spectra of a permanganate-based colorimetric assay in the presence of RCp NDs and H_2_O_2_ as reference. The absorbance reading at 652 nm for •OH triggered TMB oxidation in the presence of (c) varying H_2_O_2_ concentration and (d) Ru: Cu ratio (x = 0, 0.25, 0.50, 0.75, 1.00). (e) pH variation utilized by 100 ppm of RCp NDs, (f) RCp NDs concentration utilized at pH 5.5. GSH depletion property; (g) Relative absorption reading at 412 nm for GSH reduction monitored by Ru_*x*_Cu_1–*x*_O_2_ (x = 0, 0.50, 1.00). A control experiment was done by excluding material. Absorbance spectra for GSH solution mixed with DTNB obtained at different (h) time intervals and (i) concentrations of RCp NDs. 0.2 mM GSH and 0.4 mM DTNB were used in this experiment, (SD, *n* = 3). (j) Possible RCp NDs-based catalytic loop-mediated reactions.

The formation of peroxo groups was detected through a permanganate (MnO_4_^–^) based colorimetric analysis in the RCp NDs. The results presented in Figures 2a and 2b demonstrate that the presence of RCp NDs causes the color of permanganate (MnO_4_^–^) in an acidic solution to vanish, a phenomenon attributed to the reduction of MnO_4_^–^ to colorless Mn^2+^ due to peroxo groups produced in the system. The disappearance of the pink color (Figure 2a) and the decrease in absorption peaks in 450–580 nm (Figure 2b) confirmed the H_2_O_2_-self-producing capability of RCp NDs, as similar to the reference standard, H_2_O_2_. The •OH producing capability of RCp NDs was assessed through a Fenton (like) reaction between RCp NDs and TMB without and with different amounts of H_2_O_2_ using the TMB assay (Figure 2c). In principle, the TMB solution, after reacting with RCp NDs, oxidized by highly active •OH species and resulted in blue solution of oxTMB with at λ_max._ = 652 nm (Figure S7a).

Figure 2d demonstrates that the synergistic combination of Ru and Cu (in ratios ranging from 0.25 to 0.50) enhances •OH generation, whereas Ru alone did not have a noticeable effect on the oxTMB formation. The peroxidase activity of RCp NDs was further manipulated by varying the pH values (Figure 2e), and the dynamics of •OH generation were studied by varying concentrations of RCp NDs (Figure 2f). The findings validate the exceptional catalytic efficacy of Ru^2+^/Cu^+^ in a Fenton (like) reaction, as well as the proficient creation of •OH by Ru^3+^/Cu^2+^ for anticancer therapy. The route of H_2_O_2_-self-produced for CDT, cocatalytic properties, and the simultaneously GSH-reducing properties of NDs are explained in Figure 2j. The current work focuses on the Fenton-mediated reaction of RCp NDs, giving strong evidence in support of the hypothesis that maintaining mixed valence ratios of Cu^+^ to Cu^2+^ and Ru^2+^ to Ru^3+^ were 0.64 and 1.12 respectively (Table S1), within the Cu–Ru cocatalytic loop is critical to boost the •OH formation (Figure 2j), which unique combination allows for the simultaneous depletion of GSH in the substrate and the dissociation of H_2_O_2_ into •OH via a Fenton (like) reaction.

**Figure 3:**
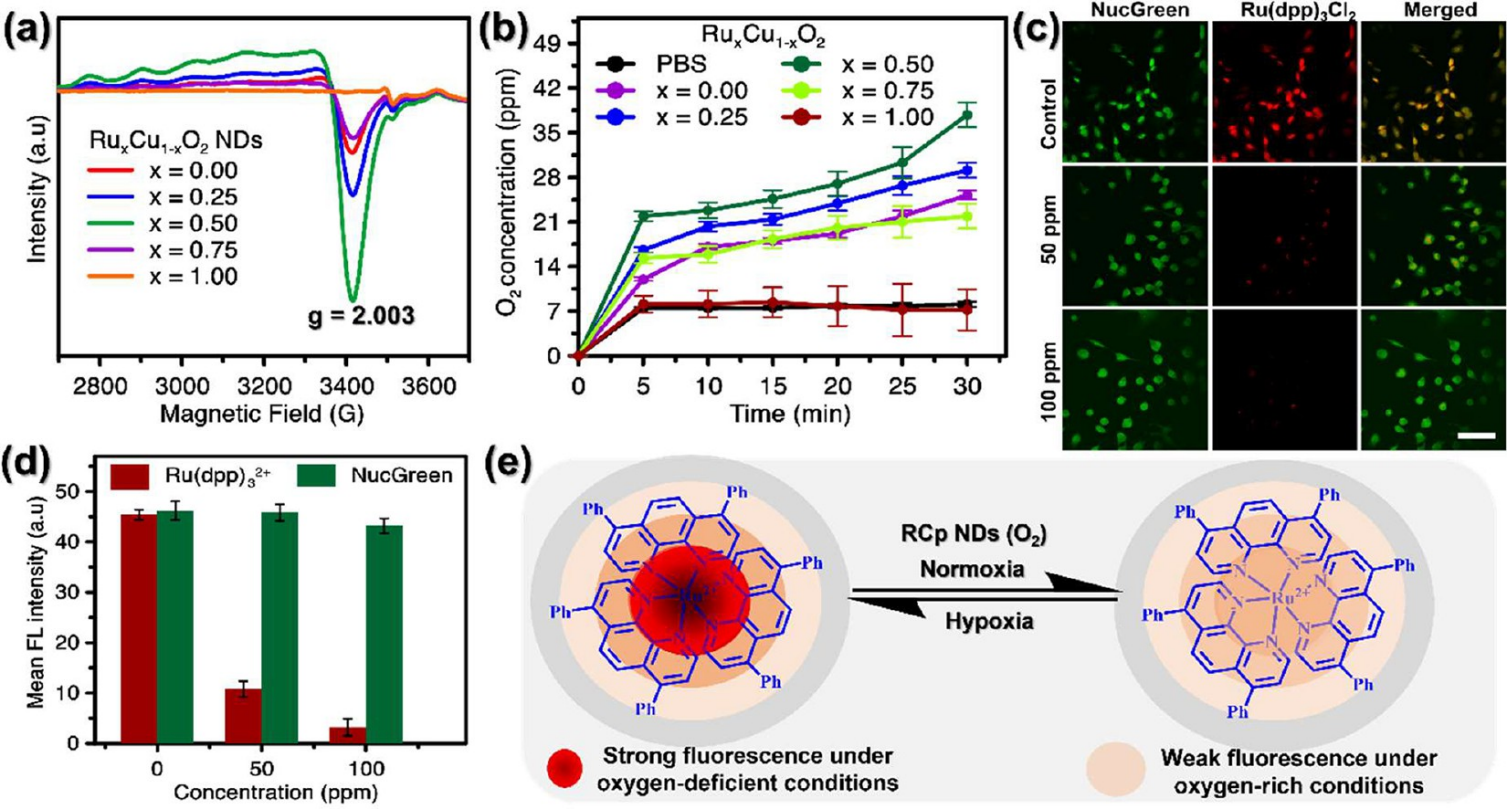
Formation of oxygen vacancy and O_2_ generation using RCp NDs: (a) ESR signal of oxygen vacancy formation at g = 2.003. (b) O_2_ generation levels in RCp NDs under various circumstances. (c) O_2_ detection using fluorescence images generated in intracellular cells. (d) The mean fluorescence intensity derived from (c) using ImageJ software. (e) Schematic illustration of an O_2_ level sensor operating in normoxia or hypoxia, with an on/off switch for luminescence emission using the Ru(dpp)_3_^2+^ probe.

Furthermore, as demonstrated in Figure 3a, ESR signal detection at g = 2.003 was applied to Ru_*x*_Cu_1–*x*_O_2_ NDs (x = 0.0, 0.25, 0.50, and 1.00), indicating that it possesses the highest O_V_ density, which grants the potential to form massive Lewis’s acid sites for peroxide adsorption.^41,42^ Then, the concentrated peroxo groups (O–O), contributing to the self-supply of H_2_O_2_ in the Fenton reaction and promoting •OH formation in the process were detected by the ESR spectra of RCp NDs, confirming the optimal bimetallic ratio, which either adsorbs or coordinates with O–O correspondingly through the O_V_.^52,53^ The stronger signal in the ESR spectra of RCp NDs (x = 0.50) in comparison to Ru_*x*_Cu_1–*x*_O_2_ NDs (x = 0.0 and 1.00) implies that the Cu–Ru ratio etching the formation of O_V_, which leads to an increased amount of H_2_O_2_ coordinated through the O_V_ on the surface of this nanodots. This structural analysis reveals that the disordered structure and the presence of O_V_ trigger interfacial redox reactions in the Cu–Ru heterogeneous system, as well as increased incorporation of H_2_O_2_ and the transfer of excess electrons in the Cu–Ru heterogeneous system.^42,54,55^ As shown in Figure 2e, RCp NDs exhibit an intense ESR signal at a g-factor of 2.003, corresponding to the unpaired electrons trapped in O_V_. In contrast, a much weaker signal is detected for Ru_*x*_Cu_1–*x*_O_2_ NDs (x = 0.0) whereas stronger signal detected for RCp NDs. The O_V_ density for RCp NDs is calculated to be 77.78%, which is more than 3.5 times that for x = 0.0, where x = 1.00 did not show any O_V_, as shown in Figure 3a.

### 3.3 GSH Depletion Property

RCp NDs can potentially boost GSH depletion activity due to the presence of two cocatalytic pairs of Ru^3+^/Cu^+^ and Ru^2+^/Cu^2+^, as demonstrated in Figure 2g. In TME, powerful intracellular antioxidant GSH, scavenges ROS produced intracellularly, causing an enhanced hypoxic environment and decreasing the effectiveness of ROS-based therapies.^44,56,57^ The capability of RCp NDs to deactivate the GSH reagent was investigated using a DTNB probe in a mildly acidic environment. The Ru^3+^/Cu^2+^ ions are reduced by GSH in the presence of the DTNB probe, resulting in the formation of a yellow TNB intermediate (Figure S7b). This intermediate is a fragment of GSSG and exhibits a distinctive absorption band at 412 nm (Figure 2j).^58,59^ As, we related the GSH depletion assets of Ru_*x*_Cu_1–*x*_O_2_ NDs (x = 0.0, 0.50, and 1.00) (Figure 2g), it confirmed that the Ru^3+^/Cu^2+^ combination enhances the overexpressed GSH depletion. Due to the lack of Ru^3+^, we found that Ru_*x*_Cu_1–*x*_O_2_ NDs (x = 0.0) showed moderately less activity toward GSH reduction than RCp NDs. In contrast, the GSH level did not show any change for 90 min in the absence of the NDs, but at x = 0.0 and 0.50 showed 48% and 80% GSH reduction from the initial concentration, respectively, (Figure 2g). Figure 3h showed a decrease in the absorption spectra of 5-thio-2-nitrobenzoic acid (TNB - intermediate) at 412 nm as the incubation period advanced, indicating that the amounts of GSH were successfully lowered by RCp NDs. The experiment was replicated by altering the ratio of RCp NDs. The results displayed in Figure 2i demonstrated an immediate decrease in the absorption spectra as the concentration of the material rose. This suggests that GSH consumption was significantly enhanced at higher concentrations of RCp NDs. Overall, the combination of Ru^3+^ and Cu^2+^ in RCp NDs significantly upgraded the GSH-depleting property, revealing credibility for improving ROS-grounded activity by deactivating the antioxidant GSH in intracellular cells.

### 3.4 Intracellular O_2_ Generation

RCp NDs exhibit exceptional O_2_ generating capability, as evidenced by comparing the time-dependent O_2_ generation profile of Ru_*x*_Cu_1–*x*_O_2_ NDs (x = 0.0, 0.25, 0.50, 0.75, and 1.00) with a buffer solution (Figure 3b). RCp NDs demonstrate superior O_2_ generation efficiency among the NDs tested. In a TME-like hypoxic environment, O_2_ is produced when H_2_O_2_ breaks apart independently, as shown in Figure 2j. This ability of RCp NDs to generate O_2_ intracellularly was assessed using Ru(dpp)_3_Cl_2_, which interacts with the O_2_ formed due to the dissociation of peroxide in the system, as illustrated in Figure 2j and generated by RCp NDs (Figure 3c). The Ru(dpp)_3_Cl_2_ complex, with λ_exc._= 561 nm and λ_em._ = 620 nm, is commonly employed for oxygen detection because its fluorescence is significantly reduced by molecular oxygen through dynamic quenching.^60,61^ Cancer cells were treated with RCp NDs and Ru(dpp)_3_Cl_2_ and then imaged using confocal spectroscopy. The fluorescence images in Figure 3c demonstrate that untreated control cells exhibited strong red fluorescence from Ru(dpp)_3_Cl_2_. In contrast, the red fluorescence intensity decreased in cells exposed to RCp NDs due to fluorescence quenching caused by oxygen production (Figure 3d). The decrease in fluorescence intensity became more pronounced with increasing quantities of RCp NDs, indicating elevated levels of intracellular oxygen production,^62^ which act as Ru(dpp)_3_^2+^ quenchers (Figure 3e). These observations underscore the effective generation of O_2_ within cells when RCp NDs break down self-produced peroxide into O_2_ molecules, as described in the reactions (Figure 2j).

### 3.5 Fabrication of RCpCCPT as Multimodal Anticancer Agent

**Figure 4:**
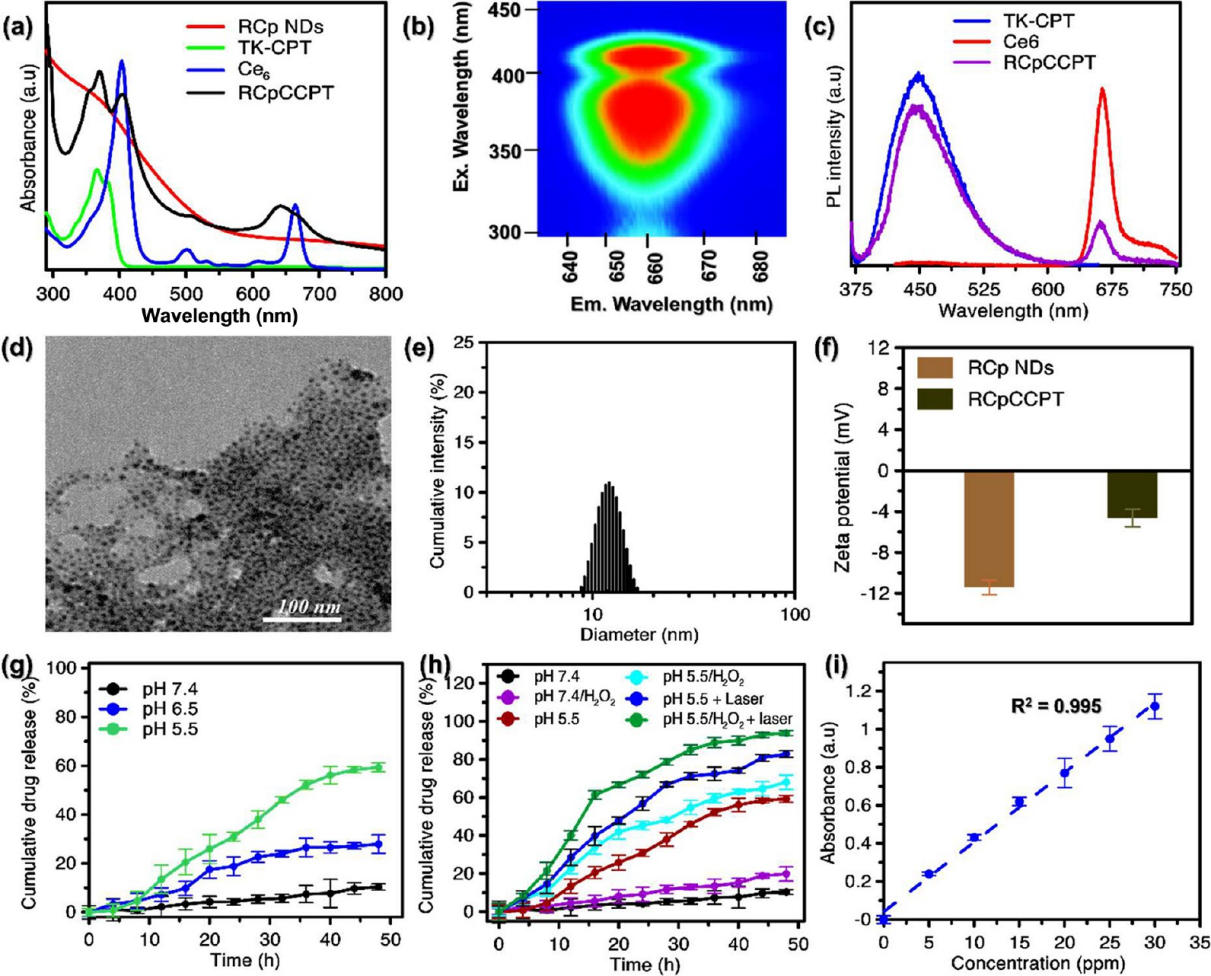
In vitro drug activity of RCpCCPT: (a) UV–visible absorption spectra of RCp NDs, TK-CPT, Ce6, and RCpCCPT. (b) 2D PLE mapping image of the RCpCCPT (c) PL emission spectra of TK-CPT, Ce6, and RCpCCPT, (d) TEM image, and (e) DLS measurement of RCpCCPT. (f) Zeta potential of RCp NDs and RCpCCPT. Drug releasing profile from RCpCCPT performed in various conditions such as (g) PBS (pH 7.4. 6.5 and 5.5), and (h) pH 7.4, pH 7.4/H_2_O_2_, pH 5.5, pH 5.5/H_2_O_2_, pH 5.5/laser, and pH 5.5/H_2_O_2_ + laser; (i) Calibration standard for CPT quantification. The data are presented as the mean ± standard deviation (SD; *n* = 3).

Fabrication of the RCpCCPT was performed in a two steps process; synthesis of ROS-responsive TK-CPT, then coupling of TK-CPT and Ce6 with RCp NDs via an esterification employing dicyclohexylcarbodiimide and 4-(dimethylamino)-pyridine (DCC/DMAP) coupling. As shown in Scheme 1, TK-CPT and Ce6 were first reacted with cross-linkers (DCC/DMAP), then conjugated with the RCp NDs using HA as a connector, resulting in RCpCCPT. The effective preparation of TK linker and TK-CPT was validated by ^1^H NMR spectra and FTIR spectrum (Figure S1 and S2). Absorption spectrum and fluorescence emission measurements were used to investigate the successful loading of drug molecules in RCpCCPT. UV–vis absorption spectra of RCpCCPT demonstrated intensive band arrangement in between 300–800 nm wavelength region (Figure 4a). The characteristic peaks at 400 and 645 nm correspond to the Ce6, which is consistent with pure Ce6 molecules. Furthermore, the peak around 366 nm is CPT, and the findings are consistent with pure CPT-TK, confirming the production of RCpCCPT (Figure 4a). The 2D PLE Mapping pictures reveal two distinct centers of excitation wavelengths, corresponding to CPT (360 nm) and Ce6 (410 nm), indicating the existence of two distinct fluorophores in the RCpCCPT solution (Figure 4b). Furthermore, broad PL emission spectra at 460 nm and weak emission at 670 nm were observed from CPT and Ce6, indicating that RCpCCPT was effectively produced (Figure 4c). As shown in Figure 3d, dot-like morphology was observed for RCpCCPT, and the size is estimated to be 7.5 nm. The hydrodynamic diameter of RCpCCPT was recalculated to 11.2 nm based on DLS results (Figure 4e), and the value was found to be greater than the size observed in TEM images (Figure 4d). This might be due to the particle growth of HA-coated RCp NDs and a layer of CPT/Ce6 on the surface of RCpCCPT. The zeta potential technique was used to analyze the charge distribution on the surface of the RCp NDs and RCpCCPT. A lower negative charge was obtained for RCpCCPT, confirming the loading of CPT/Ce6 through the hydroxyl of HA-coated RCp NDs (Figure 4f). Similarly, RCpCCPT surface functional groups were examined using FTIR. The study showed stretching peaks at 3400 cm^–1^ for COOH, 3236 cm^–1^ for = C–H (sp^2^), and 1720–1650 cm^–1^ for carbonyl. Additionally, Cu–O and Ru–O bonds were found at 640 and 500 cm^–1^ (Figure S5). RCpCCPT CPT loading content and efficiency were measured using UV–vis absorption spectroscopy (Figure 4i). The results were 38.1% and 79.3%.

### 3.6 ROS-Based Drug Release

It is possible to combine anticancer treatments with the development of a prodrug platform using ROS-responsive TK linkage drug delivery technique.^45,63^ This allows the release of drugs when ROS is generated, which can address challenges related to drug delivery and distribution in tumor therapy.^45,64,63^ As discussed in section 3.2, RCpCCPT has demonstrated the ability to generate ROS (•OH, ^•^O_2_^–^) in an acidic environment and can additionally produce ^1^O_2_ when exposed to laser light. ROS production can potentially induce the fast rupture of the TK link, as depicted in Scheme 1. The release of CPT from RCpCCPT was investigated under various pH conditions and in the presence of external stimuli such as laser, which could induce significant ROS production and trigger the breakdown of the TK bond for efficient drug release (Figures 4g and 4h). In the absence of external stimuli but with varying pH values, RCpCCPT released approximately 58.5% of CPT at pH 5.5, thanks to the self-production of H_2_O_2_ by RCpCCPT in an acidic environment. Lower drug release values were observed at pH 6.5 (24%), and less than 10% at pH 7.4 (Figure 4g) within 48 h. However, upon activation of the reaction with external stimuli such as H_2_O_2_ and laser, the cumulative release of CPT reached up to 70% and 86%, respectively, due to the significant formation of ROS through the Fenton reaction and light-induced ^1^O_2_ (Figure 4h). Remarkably, considerable drug release was achieved in an acidic environment without external stimuli, owing to the self-supply of H_2_O_2_ in the TME (Scheme 1). This makes our material more suitable for in vivo applications.

### 3.7 ROS-Based Therapies of RCpCCPT

**Figure 5:**
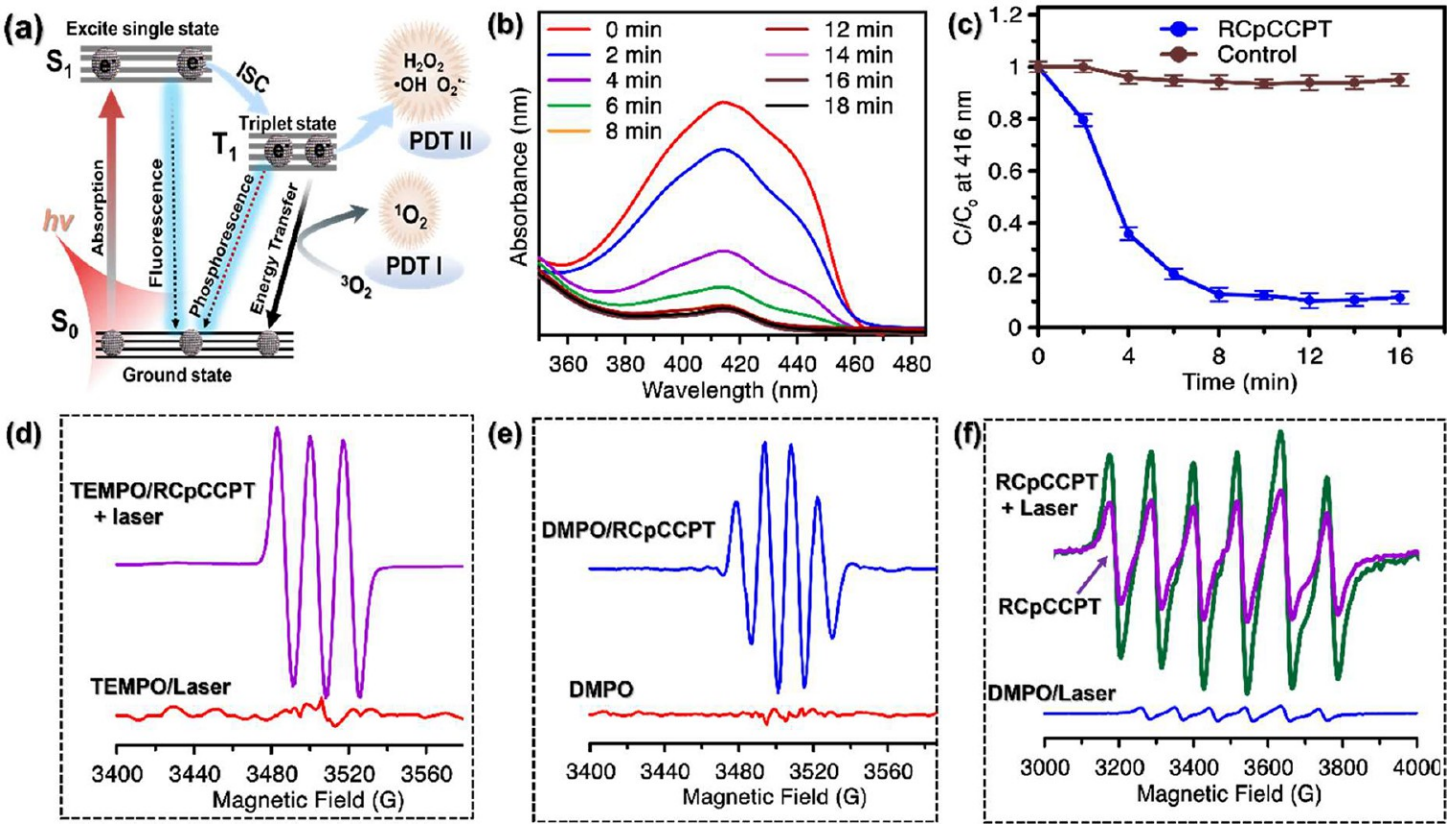
Mechanism of ROS generation and detection of RCpCCPT: (a) Schematic illustration of TME-mediated synergistic Fenton reaction by RCpCCPT for enhanced CDT with self H_2_O_2_-production; hypoxia-relief via sufficient oxygen supply for photonics-induced ROS-generating activity. (b) UV–vis absorption spectra of 1.0 mM DPBF solution containing 100 ppm RCpCCPT treated with laser light at various times, (c) Comparison for normalized absorption intensity of DPBF solution (at λ = 416 nm) obtained with and without RCpCCPT treated using a laser light (SD, *n* = 3). ESR spectroscopic analysis of (d) 4-oxo-TEMP signals of ^1^O_2_ in the presence and absence of RCpCCPT with laser illumination, (e) ESR signal DMPOO• responding to •OH formation in the presence and absence of RCpCCPT solution, and (f) ESR signal DMPOO/DMSO solution for responding to the generation of ^•^O_2_^–^ in RCpCCPT with (green) and without laser (pink). All laser illumination experiments were conducted with a 671 nm, 1.0 W/cm^2^ laser source.

As depicted in Figure 5a, RCpCCPT shows excellent potential as a ROS generator when exposed to light irradiation. This makes it a promising photosensitizer for light-activated therapeutic applications and a multi-ROS producer by decomposing H_2_O_2_ and breaking of O_2_, such as •OH, ^•^O_2_^–^, or ^1^O_2_. To ensure the reliability and precision of our results, we employed specific DPBF probes to detect ^1^O_2_. When ROS interacts with the DPBF probe, the absorption of molecules is reduced at 416 nm, and it transforms into colorless 1,2-dibenzoylbenzene (DBB).^65^ This phenomenon was confirmed by observing the UV–vis absorption spectrum at 416 nm. As shown in Figure 5b, the absorption spectrum of DPBF gradually diminished with increasing light incubation period in the presence of RCpCCPT, underscoring the effective ROS-generating properties of RCpCCPT. No noticeable absorbance change of DPBF was identified when only laser irradiation was utilized, but more than 80% absorbance of DPBF was reduced in the presence of RCpCCPT (Figure 5c). Thus, the hypothesis suggests that RCpCCPT is designed to be self-sufficient regarding H_2_O_2_ and O_2_, overcoming hypoxia conditions and enabling incredibly effective PDT in solid tumors. ROS are primarily produced as a result of light activation by two distinct processes: an electron-transfer reaction that splits water or H_2_O_2_ into •OH and ^•^O_2_^–^ (PDT II); and an energy-transfer reaction that catalyzes the formation of ^1^O_2_ from molecular O_2_ (PDT I), increasing the generation of ROS.

ESR spectroscopy was employed to investigate the ROS-producing capacity of RCpCCPT qualitatively. In the present study, two distinct probes, TEMP and DMPO, were used to detect ^1^O_2_ and •OH/^•^O_2_^–^), respectively. When RCpCCPT was incubated with TEMP under laser irradiation, TEMP generated a characteristic set of TEMP-1-Oxyl (TEMP) signal peaks in a 1:1:1 ratio. This observation became notably more prominent, signifying the production of ^1^O_2_ (Figure 5d). Similarly, as depicted in Figure 5e, the DMPO/H_2_O_2_ solution did not yield any signals, while DMPO/RCpCCPT in water solutions exhibited quartet signal peaks in a 1:2:2:1 ratio. The observed outcome can be attributed to the generation of DMPOO- adducts, which occurred as a consequence of •OH produced via the Fenton (like) reaction. This finding confirms the presence of self-produced H_2_O_2_ in RCp NDs, which contributes to the Fenton reaction in an acidic environment and the self-induced formation of •OH. Likewise, ^•^O_2_^–^ formed within the system, facilitated by the presence of self-produced H_2_O_2_ and influenced by the Ru/Cu pair, was detected through the ESR spectrum. As indicated in Figure 5f, ESR spectra of DMPO/RCpCCPT in DMSO solutions displayed signal peaks in a 1:1:1:1:1 ratio, indicating the formation of DMPOO-O_2_ adducts due to ^•^O_2_^–^ production. This ^•^O_2_^–^ production results from H_2_O_2_ dissociation catalyzed by the bimetallic interaction in an acidic environment, as depicted in Figure 2j. In summary, ESR spectroscopy and colorimetric assays confirmed that RCpCCPT predominantly generated ^1^O_2_, •OH, and ^•^O_2_^–^ within the system. A schematic illustration, as depicted in Scheme 1, was proposed to elucidate the mechanism behind effective ROS generation by RCpCCPT and the multiple exciton properties of RCpCCPT.

### 3.8 In Vitro Biocompatibility Studies of RCp NDs/RCpCCPT

**Figure 6:**
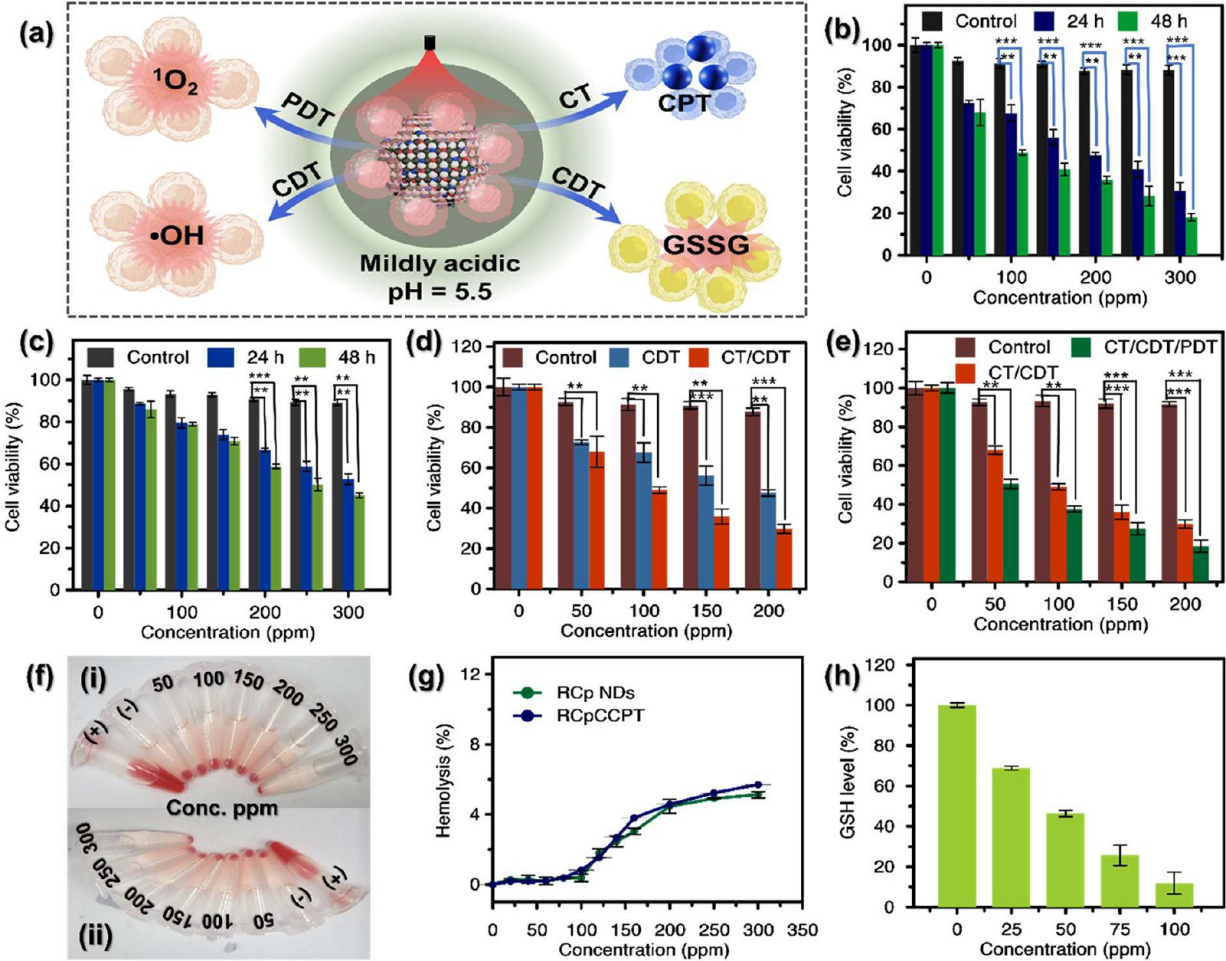
Intracellular biocompatibility experiment: (a) Schematic mechanism for RCpCCPT-induced cancer cell apoptosis at the mildly acidic environment. Cellular viabilities of (b) MDA-MB-231 and (c) 4T1 cells incubated with various concentrations of RCp NDs (0–300 ppm) for 24 and 48 h. (d) In vitro CT and CDT/CT evaluation using MDA-MB-231 cells incubated with different concentrations (0–200 ppm) of RCp NDs (for CDT) or RCpCCPT (for CT/CDT). (e) In vitro CDT/CT and CDT/CT/PDT evaluation using MDA-MB-231 cells incubated with different concentrations (0–200 ppm) of RCpCCPT without and with laser irradiation. (f) Hemolytic assay of RCp NDs (i) and RCpCCPT (ii) with mouse red blood cells (RBCs). The treatment of red blood cells (RBCs) with either Triton X-100 or PBS buffer was used in negative (−) and positive (+) controls. (g) Hemolytic assay of RCp NDs and RCpCCPT determined by measuring the absorbance at 576 nm. (h) Intracellular GSH reduction performance was detected at 412 nm by varying the concentrations of RCpCCPT. The data are presented as the mean ± standard deviation (SD; *n* = 3). Significant differences at the same drug concentration at *p < 0.05, **p < 0.01, ***p < 0.001 are indicated.

Motivated by the outcomes of the aforementioned extracellular tests, RCpCCPT is anticipated to trigger apoptosis due to PDT, CDT, CT, and GSH depletion. In Figure 5a, the potential graphic approach is shown. Because the moderate acidic behavior of TME can increase the ability to generate •OH and encourage the self-disintegration of RCpCCPT, more surface flaws are exposed, leading to a more significant Fenton (like) reaction rate. Next, Figure 6a shows how cancer cell lines were subjected to the synergistic CDT/CT/PDT impact. In our cytocompatibility experiments, we utilized MDA-MB-231 and 4T1 cells to assess the effect of Ru–Cu peroxo group of RCp NDs compared to Ru–Cu oxides for intracellular studies. As depicted in Figures S8a and S8b, the MDA-MB-231 and 4T1 cell lines, respectively, were subjected to varied doses of Ru–Cu oxides (0–300 ppm) for varying durations (24 and 48 h). The results of WST-1 assays confirmed that both MDA-MB-231 and 4T1 cells maintained high viability, with cell percentages exceeding 90%, even when exposed to a concentration as high as 300 ppm of Ru–Cu oxides for 48 h. Nevertheless, the findings depicted in Figures 6b and 6c demonstrate that the RCp NDs displayed the capacity to eliminate cancer cells when subjected to different doses and durations of incubation. The incubation of MDA-MB-231 and 4T1 cancer cell lines at a concentration of 300 ppm of RCp NDs for 48 h resulted in a decrease in cell viability of up to 18% and 48% respectively. The decrease in cancer cell viability depicted in Figures 6b and 6c can be ascribed to the self-generated Fenton (like) reaction, which produces •OH within the system, commonly referred to as the CDT effect.

Additionally, we evaluated the impact of RCp NDs and RCpCCPT on blood through a hemolysis assay using mouse blood cells. Ensuring hemocompatibility is of utmost importance for nanomaterials, as it ensures their safety in biomedical applications, especially when they come into contact with blood.^46,47^ As illustrated in Figures 6f and 6g, neither RCp NDs nor RCpCCPT exhibited any detrimental effects on red blood cells (RBCs) at the concentrations tested, as compared to the positive controls. As illustrated in Figure 6g, the results indicate that the hemolysis percentages remained below 5.5%, even at higher concentrations. This highlights the exceptional hemocompatible properties of both RCp NDs and RCpCCPT, making them highly suitable for various biomedical applications.^46,47^

### 3.9 Intracellular Activity of RCp NDs and RCpCCPT

As can be noticed Figures 6d and 6e, highlight the efficacy of extracellular therapeutic applications of RCpCCPT as a potent anticancer platform that combines CDT and CT, with the added potential for PDT when exposed to a light source. This reduction can be credited, to when laser light was applied as an external stimulus, over 81.5% of the cells were eradicated (Figure 6e), thanks to the synergistic effects of CT/CDT/PDT. These results confirm that cancer cells effectively internalize RCpCCPT, which subsequently generates destructive ROS within the cell when exposed to the TME. RCpCCPT acts as H_2_O_2_ self-supplying nanocarrier, and this effect is amplified when there are outside influences present, like light sources.

Additionally, RCpCCPT’s redox capabilities can reduce intracellular GSH content while elevating ROS levels, leading to significant oxidative stress in tumor cells. To quantify the reduction in GSH levels, MDA-MB-231 cells were exposed to several concentrations of RCpCCPT overnight, as depicted in Figure 6h. The intracellular GSH levels decreased with increasing RCpCCPT concentration. Notably, after incubation with 100 ppm RCpCCPT, 95.6% of the intracellular GSH levels were depleted, related with the control group (Figures 6h and S9). This indicates that RCpCCPT has significant potential as an antioxidant deactivator, enhancing ROS-mediated therapeutics in the field of nanomedicine.

### 3.10 Mechanism of Intracellular and Endocytosis Uptake

**Figure 7:**
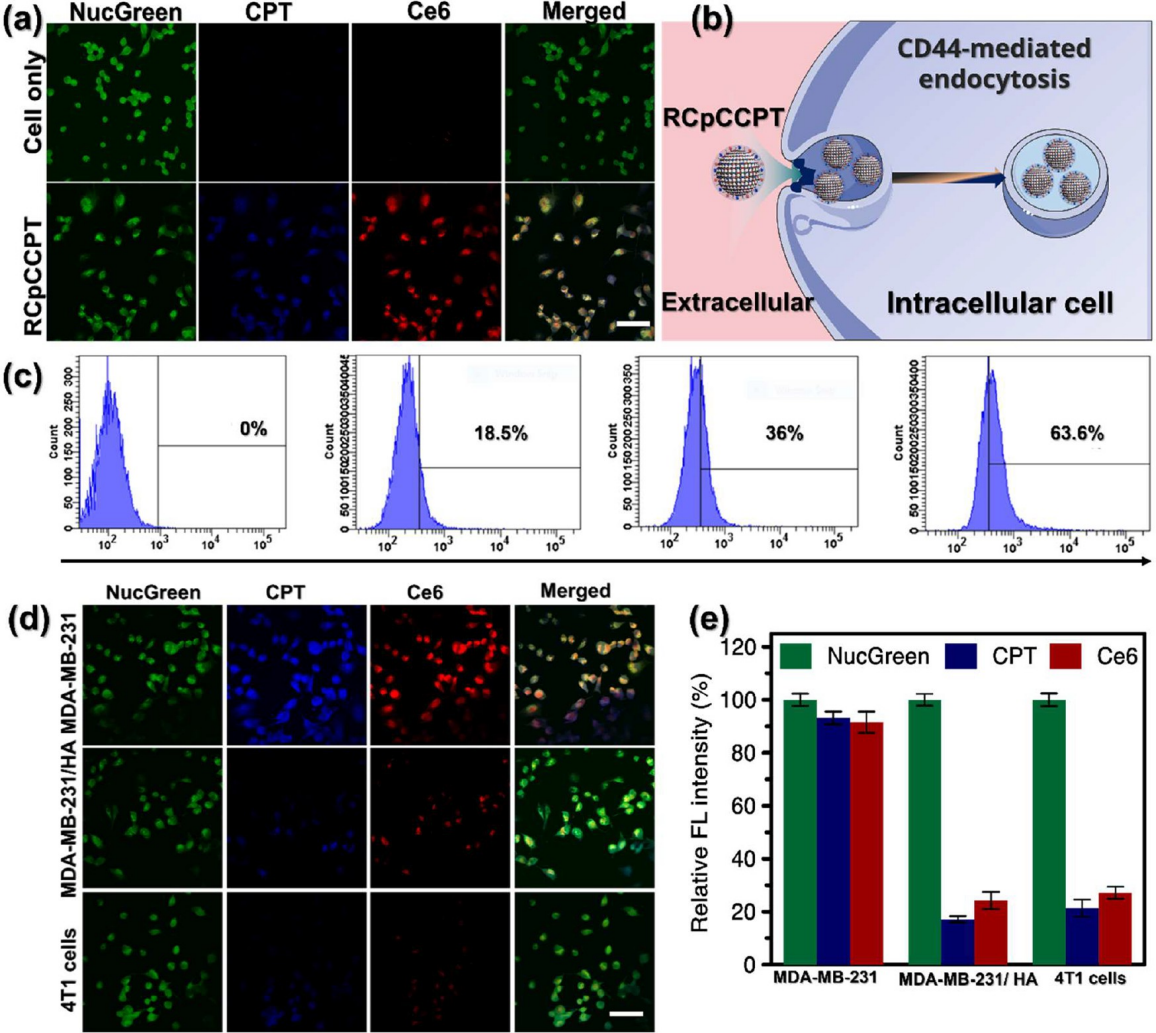
Intracellular uptake experiment: (a) Confocal micrography of MDA-MB-231 cells stained with NucGreen and incubated with RCpCCPT. Green fluorescence indicates the nucleus, blue and red fluorescence corresponds to RCpCCPT, and merged pictures reveal three fluorescence superimposed. The scale bar is 50 μm. (b) Cellular uptaking mechanism of RCpCCPT via endocytosis trafficking in an intracellular cancer cell. (c) Cellular uptake confirmation through the Flow cytometer technique, the experiment was performed using increasing RCpCCPT concentration from left to right (0, 25, 50, 75, 100 ppm). (d) Confocal fluorescence images of MDA-MB-231 and 4T1 cells treated with RCpCCPT and labeled with NucGreen. Confocal fluorescence image of MDA-MB-231/HA group shows that MDA-MB-231 cells were exposed to 100 μg/mL free HA for 3 h. Subsequently, the cells were then treated with RCpCCPT at 37 °C for 24 h. Using green light, the NucGreen channel tags cell nuclei. The blue and red emissions channels represent RCpCCPT’s position, whereas overlay imaging shows all three. The scale bar is 50 μm. (e) Relative FL intensity of different fluorescent signals taken from Figure (d).

Intracellular and endocytosis trafficking of RCpCCPT were assessed using MDA-MB-231 cells treated with RCpCCPT overnight. The treatment involved using RCpCCPT in conjunction with a nuclear probe, as depicted in the microscopic images in Figure 7a. The associated endocytosis mechanism is illustrated in Figure 7b. In these images, the blue/red emissions from RCpCCPT are observed through the pinocytosis-mediated endocytosis mechanism of MDA-MB-231 cells, while the green emission arises from NucGreen labels the locations of cell nuclei. The blue fluorescence corresponds to CPT emission, while the red fluorescence corresponds to Ce6 emission. These observations confirm the successful endocytosis process through which RCpCCPT was absorbed into the cells. Furthermore, we used flow cytometry to evaluate the uptake of RCpCCPT by cells. A control group and various concentrations of RCpCCPT (0, 50, 75, and 100 ppm) were added. In comparison to the control, the results showed a promising intake of RCpCCPT and a robust dose-dependent response by the cells (Figure 7c). It should be highlighted that 63.6% of the RCpCCPT was uptaken by the cells. This suggests a high level of cellular uptake, which is the cause of apoptosis. Additionally, to confirm the specificity of RCpCCPT binding to CD44 receptors, we conducted a comparison experiment using MDA-MB-231 cells, which are rich with CD44 receptors, and 4T1 cells, which are deficient with CD44 receptors.^66,67^ The results, depicted in Figure 7d, revealed that 4T1 cells exhibited minimal internalization of RCpCCPT, displaying only scattered punctate blue-red fluorescence in the cytoplasm compared to MDA-MB-231 cells. This suggests that 4T1 cells, deficient with CD44 receptors on their membrane surface, are unable to internalize RCpCCPT to the extent observed in MDA-MB-231 cells. Furthermore, to confirm that RCpCCPT uptake primarily occurs through CD44 receptor-mediated endocytosis, we conducted competition experiments by incubating RCpCCPT with MDA-MB-231 cells in the absence or presence of excess free HA, which blocks HA receptors. Our findings indicated a significant decrease in RCpCCPT uptake by MDA-MB-231 cells in the presence of free HA, with minimal fluorescence detected (Figure 7d). This implies that excess free HA competes for surface receptors on MDA-MB-231 cells, thereby hindering the intracellular uptake of RCpCCPT. The relative fluorescent intensity calculated in Figure 7e also strengthens the dominance of RCpCCPT uptake through the CD44-endocytosis mechanism compared with the CD44-deficient 4T1 cells and MDA-MB-231 cells preblocked with HA receptors. These results strongly support the notion that RCpCCPT uptake is indeed associated with CD44 receptor-mediated targeted delivery.

**Figure 8:**
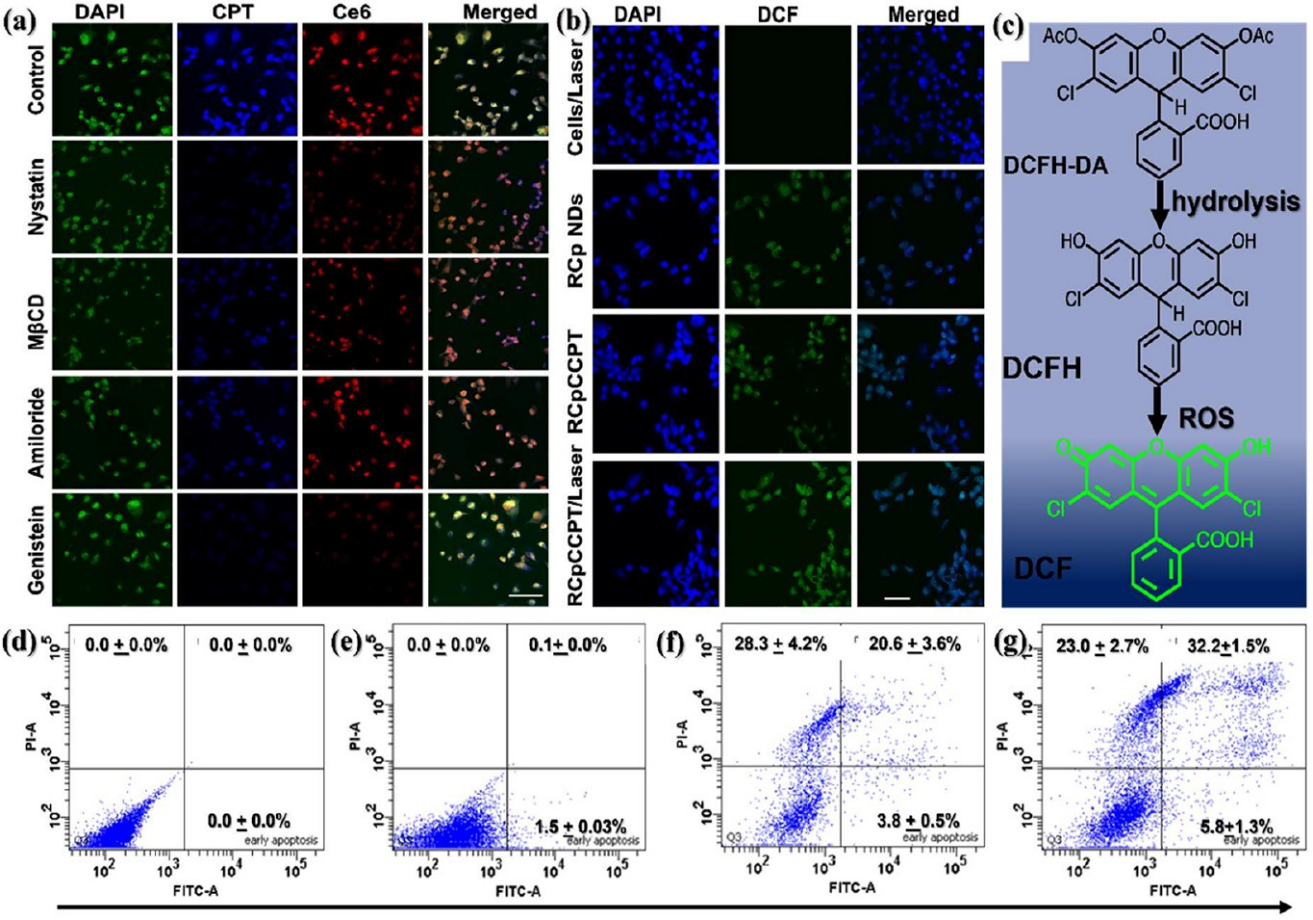
Endocytosis uptake mechanism: (a) Cellular imaging of MDA-MB-231 cells incubated with 45 μL inhibitors (nystatin, genistein, amiloride, and MβCD) for 30 min, then treated with 100 ppm of RCpCCPT for 3 h. The control experiment was performed without adding an inhibitor. The blue, green, and red channel images were captured using 405, 488 nm, and 561 excitation wavelengths, respectively. The merged images show superimposed images of blue, green, and red fluorescence. (b) Confocal images of MDA-MB-231 cells incubated with cells with laser as control, RCp NDs, RCpCCPT, and RCpCCPT/laser for 12 h at 37 °C in a humidified atmosphere. The cancer cells were stained with DAPI to track the nucleus. The green channel indicates that the DCFH-DA has been oxidized into DCF species after reacting with the ROS generated in the cellular organ system. The scale bar is 50.0 μm. (c) Hydrolysis and ROS-induced oxidation of DCFH-DA with RCpCCPT results in the formation of DCF, green fluorescent material. Flow cytometry analysis of apoptotic MDA-MB-231 cells after various treatments; (d) cells only, (e) cells treated with laser, (f) RCp NDs (CDT), and (g) synergistic effect, RCpCCPT/Laser (CT/CDT/PDT) incubated with cells. Experiments requiring irradiation were carried out using a 671 nm laser source (1.0 W/cm^2^) for 5 min while data represents ± SD, *n* = 3.

Additionally, Figure 8a illustrates the mechanism of intracellular uptake by utilizing various inhibitors. In this experiment, MDA-MB-231 cell lines were subjected to a 30 min preincubation with inhibitors, including amiloride, genistein, nystatin, and methyl-β-cyclodextrin (MβCD). Following this preincubation, the cells were then exposed to 100 ppm of RCpCCPT for 3 h. The CLSM pictures presented in Figure 8a demonstrate that the inhibitors genistein and nystatin effectively impede the uptake of RCpCCPT. This observation suggests that the process of pinocytosis-mediated endocytosis is a plausible mechanism for the internalization of RCpCCPT. As a result, MβCD inhibitors facilitated less RCpCCPT absorption compared to the amiloride inhibitor, which exhibited similar mean fluorescence intensity to the control experiment. This result suggests that RCpCCPT is CD44-endocytosed via the pinocytosis pathway,^68^ as depicted in Figure 7b.

### 3.11 Intracellular ROS Detection

The ability of RCpCCPT to induce intracellular oxidative stress under external stimuli was investigated by detecting intracellular ROS using a DCFH-DA reagent. DCFH-DA is a nonfluorescent molecule that interacts with ROS produced within cellular organelles to produce green fluorescence known as 2′,7′-dichlorofluorescein (DCF), as illustrated in Figures 8b and 8c. DAPI was utilized to stain the cell nuclei. The CLSM images in Figure 8b show that MDA-MB-231 cells treated with laser/H_2_O_2_ alone did not exhibit a green fluorescence signal. In contrast, the cells treated with RCp NDs and RCpCCPT exhibited more pronounced green fluorescence signals than the cells in the control group. This suggests the intracellular generation of •OH through Fenton reactions. Furthermore, higher green fluorescence intensities were observed when combined with a light source as an external stimulus for ^1^O_2_ initiation (Figure 8b). This increase in fluorescence is attributed to the excess •OH, ^•^O_2_^–^, and ^1^O_2_ produced in the cell’s organ, which promotes the formation of DCF (Figure 8c). These findings confirm that RCpCCPT can generate ROS through either Fenton reactions or exposure to a light source, and the extent of ROS generation depends on the RCpCCPT concentration and external stimulus factors.

### 3.12 Cell Apoptosis and In Vitro Fluorescence Imaging of Therapeutic Assay

The Annexin-FITC-A/PI double labeling assay was employed in the cell apoptosis experiment to assess whether MDA-MB-231 cells underwent apoptosis in response to various treatments. There was no noticeable cell death when the cells were exposed to H_2_O_2_ alone as a control (Figure 8d). Similarly, H_2_O_2_/laser alone did not induce significant cell apoptosis (Figure 8e). However, when cells were incubated with RCp NDs, there was evidence of 28.3 ± 4.2% necrosis, 3.8 ± 0.5% early apoptosis, and 20.6 ± 3.6% late apoptosis, indicating that RCp NDs could induce cell apoptosis through the process of CDT (Figure 8f). A more significant increase in total apoptosis (early + late apoptosis) was observed, reaching 55.2 ± 4.2%, when MDA-MB-231 cells were treated with RCpCCPT in combination with a laser source (Figure 8g). This outcome indicates that the efficient generation of ROS and CPT release had the potential to induce apoptosis in MDA-MB-231 cells via a cooperative effect of CDT/PDT/CT (Scheme 1). These results underscore the promising applications of RCpCCPT in cancer treatment in future nanomedicine applications.

### 3.13 Therapeutic Assessment in MDA-MB-231 Xenograft

**Figure 9:**
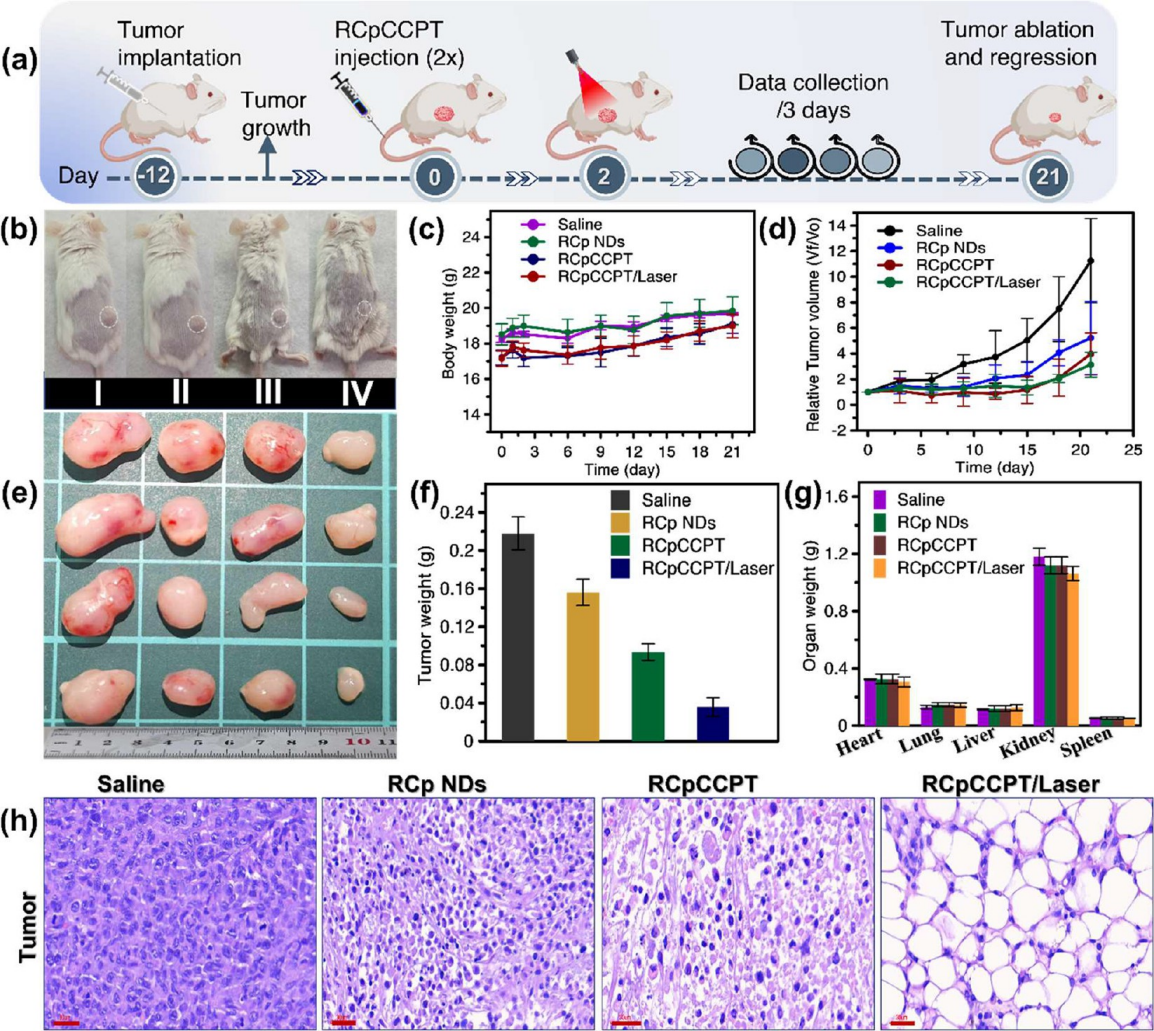
Therapeutic assessment of breast cancer xenografts: (a) Diagram for the in vivo treatment illustrations process. (b) The representative photo of MDA-MB-231 tumor-bearing mice on 21-day (where I - saline; II- RCp NDs; III-RCpCCPT; IV-RCpCCPT treated with laser). (c) Mice body weight changes after treating with different samples. (d) The relative tumor volume growth curves of tumor-bearing mice during treatments per day. (e) Representative photos of tumors harvested from tumor-bearing mice on day 21 (where I - saline; II- RCp NDs; III-RCpCCPT; and IV-RCpCCPT treated with laser). (f) Average tumor weight and (g) average organ weights harvested for tumor-bearing mice after treating with different samples on day 21. (h) H&E images of the tumor taken from tumor-bearing mice treated with saline, RCp NDs, RCpCCPT, and RCpCCPT with laser treatment. The scale bar is 60 μm. The statistics are shown as the standard deviation (±SD; *n* = 4).

Expanding on the encouraging chemodynamic efficacy observed with RCp NDs and the combined chemo/chemodynamic treatment of RCpCCPT in vitro, we evaluated their impact on inhibiting tumor growth in vivo following intravenous (i.v.) administration. For this purpose, we implemented in vivo cancer treatment at a dosage of 5 mg/kg, using the MDA-MB-231 xenograft murine model as illustrated in Figure 9a. Initially, mice were first divided into four groups (Figure 9b) with the administration of saline (I), RCp NDs (II), RCpCCPT (III), or RCpCCPT/laser (IV). Then, the MDA-MB-231 cell-derived xenograft model was established in nude mice with 1 × 10^6^ cells by subcutaneous injection. As nodules reached 50 mm^3^ in volume, mice in each group received intravenous injections of saline, RCp NDs, RCpCCPT, or RCpCCPT combined with laser exposure, as illustrated in Figure 9a. After receiving the treatments, the body weight and tumor volume of each animal was monitored and measured every 3 days, as shown in Figures 9c and 9d. Compared to animals exposed to saline, it revealed a significantly slow tumor growth rate in mice exposed to the RCp ND-treated group. Obviously, the mice that received the treatment of RCpCCPT followed by laser exposure exhibited the slowest tumor growth rate, suggesting this combined RCpCCPT plus laser exposure exerted the strongest antitumor effect. To assess therapeutic effect of each treatment, the tumors were surgically resected from mice and the nodule weight was quantified, as illustrated in Figure 9e. The results indicated the average tumor weight was 0.22 ± 0.02g, 0.16 ± 0.02g, 0.09 ± 0.01g, and 0.03 ± 0.01g in mice exposed to saline (I), RCp NDs (II), RCpCCPT (III), or RCpCCPT/laser (IV), respectively (Figure 9f). It suggested all RCp NDs, RCpCCPT and RCpCCPT/laser showed the antitumor effect in xenografts and RCpCCPT/laser exhibited the greatest inhibitory effect of tumor growth among these therapeutic candidates. These results conclusively affirmed that RCpCCPT engaged in a Fenton reaction, generating cytotoxic •OH, leading to TK breakage and subsequent release of CPT drug, which were causes for tumor growth inhibition. Upon light exposure, the RCpCCPT produces O_2_, as detailed in Section 3.4, which presents a more advanced treatment approach by addressing the hypoxic environment typically found in tumors. This augmentation served to increase the oxygen supply for PDT treatment. Consequently, the results obtained from tumor-bearing mice treated with RCpCCPT in conjunction with laser irradiation confirmed the synergistic inhibition of tumor growth via CDT/CT/PDT mechanisms. During treatments, all mice receiving i.v. injections of RCp NDs or RCpCCPT did not exhibit any significant change in body weight over the 21-day observation period (Figure 9c). No significant alterations in body weight or major organs were observed across all treatment groups (Figure 9g), confirming the biocompatibility of RCp NDs and RCpCCPT. To further assess the therapeutic outcomes, major organs, and excised tumors underwent hematoxylin and eosin (H&E) staining. Correspondingly, the results of H&E staining (Figure S10) revealed no apparent damage to the major organs compared to saline-treated mice, suggesting the good biocompatibility of RCp NDs and RCpCCPT. Figure 9h depicts a notable abundance of necrotic or apoptotic tumor cells in the RCpCCPT/laser-treated group, contrasting with relatively fewer occurrences in other groups.

On the other hand, to assess the compatibility of the material with living cells, blood samples were collected from euthanized mice and subjected to a thorough analysis of biochemical parameters. The results of the blood experiments indicated that neither RCp NDs nor RCpCCPT caused any alterations in biochemical parameters such as blood urea nitrogen (BUN), aspartate aminotransferase (ALT), and alanine aminotransferase (AST) (Table S2), and these parameters remained within the normal physiological range.^69,48^ This confirmed the biosafety of RCp NDs and RCpCCPT in tumor-bearing mice and supported their potential for future clinical investigations.

## 4 Conclusion

In this study, we successfully developed a Ru–Cu peroxide nanocarrier by surface modification with HA and coloaded with Ce6 and ROS-triggered drug release as RCpCCPT, for TME-responsive and catalytic-based multitherapeutic nanoagent. Then, we extensively studied the pH-responsive release of •OH generation and self-supply of H_2_O_2_ for Fenton (like) reactions using RCp NDs/RCpCCPT. The self-supplied H_2_O_2_ and the decomposition of Ru^2+^/Cu^1+^ trigger a Fenton (like) reaction, producing abundant •OH, depleting the GSH overexpressed, and thereby enhancing drug release in the TME via the TK linker breakage. When exposed to light, the nanocarrier can generate sufficient ^1^O_2_, even in a hypoxic environment due to the dissociation of H_2_O_2_, serving as an oxygen source. The impressive performance of RCp NDs and RCpCCPT in both in vitro studies using breast cancer MDA-MB-231 cells and in vivo experiments employing animal models showcased their remarkable high biocompatibility and capability to enhance H_2_O_2_ levels for Fenton reaction-based CDT, CDT/CT, and the synergistic Xdynamic therapy. Notably, these therapies proved to be inherently inert and selectively exhibited therapeutic effects at the tumor site, emphasizing a high level of biosafety. The entirety of these findings elucidates a meticulously designed anticancer therapeutic approach and provides insights into the potential application of RCpCCPT/laser-mediated combination therapy for the safe and effective treatment of cancers, which holds promise for future applications in biomedicine.

## Supporting Information Available

Some experimental sections, ^1^H NMR and FT-IR spectra of TK and CPT-TK, The TEM image of RCp NDs, Biosolubility and stability of RCp NDs, and RCpCCPT in different solvents, FTIR spectra of RCp NDs, and RCpCCPT, and CPT-TK, oxTMB and GSSG formation, H&E staining of main organs (PDF)
