# Supplementary material for: Engineering H2O2 Self-Supplying Platform for Xdynamic Therapies via Ru–Cu Peroxide Nanocarrier: Tumor Microenvironment-Mediated Synergistic Therapy
Source: ACS Appl Mater Interfaces. 2024 Apr 30;16(19):24172–90. doi: 10.1021/acsami.3c18888 (PMC11103653; doi:10.1021/acsami.3c18888)
Supplement: Supplementary file 1 — am3c18888_si_001.pdf [file am3c18888_si_001.pdf]

Supporting Information

Engineering H_2O_2 Self-supplying Platform for Xdynamic Therapies via Ru-Cu Peroxide Nanocarrier: Tumor Microenvironment- Mediated Synergistic Therapy

Worku Batu Direrssa[a], Tzu-Chun Kan[b], Jungshan Chang[b,c,d], Girum Getachew[a], Sonjid Ochirbat[c], Shamsa Kizhepat [a], Aswandi Wibrianto[a], Akash Rasal[a], Hung-An Chen[a], Anil Vithal Ghule[e], Tzung-Han Chou[f]*, Jia-Yaw Chang[a]*

[a] Department of Chemical Engineering, National Taiwan University of Science and Technology, Taipei, 106335, Taiwan, Republic of China.

[b] Graduate Institute of Medical Sciences, College of Medicine, Taipei Medical University, Taipei 11031, Taiwan

[c] International Master/Ph.D. Program in Medicine, College of Medicine, Taipei Medical University, Taipei 11031, Taiwan

[d] International Ph.D. Program for Cell Therapy and Regeneration Medicine, College of Medicine, Taipei Medical University, Taipei 11031, Taiwan

[e] Green Nanotechnology Laboratory, Department of Chemistry, Shivaji University, Kolhapur 416004, India.

[f] Department of Chemical and Materials Engineering, National Yunlin University of Science and Technology, Yunlin, 64002, Taiwan, Republic of China.

*Corresponding author: Tzung-Han Chou and Jia-Yaw Chang

Department of Chemical Engineering, National Taiwan University of Science and Technology, 43, Section 4, Keelung Road, Taipei, 106335, Taiwan, Republic of China

E-mail: chouth@yuntech.edu.tw (Tzung-Han Chou) and jychang@mail.ntust.edu.tw (Jia-Yaw Chang)

Tel.: +886-2-27303636.

Fax: +886-2-27376644.

The supporting information data contained as follows:

Some sections of experimental parts and a list of characterization methods used

Figure S1. ^{1}H NMR and FT-IR spectra of TK

Figure S2. ^{1}H NMR and FT-IR spectra of CPT-TK,

Figure S3. TEM image of RCp NDs

Figure S4. HRTEM and its elemental mapping images, XRD and Zeta potential of RCp NDs

Figure S5. FTIR of RCp NDs and RCpCCPT

Figure S6. RCp NDs and RCpCCPT stability in different solvent

Figure S7. The oxTMB formation at different pH-dependent and yellow colors diminishes as the incubation time of GSH + DTNB increases in the presence of RCp NDs.

Figure S8. Intracellular biocompatibility results of Ru-Cu oxides prepared without adding H_2O_2 for intracellular studies as a comparison test

Figure S9. The standard curve of optical density (OD) versus the different concentrations of GSH (recovery test $R^2 = 0.99$) using a DTNB probe at 412 nm, (SD, n =3)

Figure S10. (a) representative photo of tumor-bearing mice main organs and tumor harvested on day 21 of treatments and corresponding H&E staining.

Table S1. The Cu^+/Cu^{2+} and Ru^{2+}/Ru^{3+} ratios in RCp NDs

Table S2. Blood and biochemical data of serum analysis of the MDA-MB-231 tumor-bearing mice treated with different samples

Notes

The authors declare no competing financial interest.

Acknowledgments

The authors would like to acknowledge financial support from the National Science and Technology Council of the Republic of China (Contract No. 112-2113-M-011-002). Thanks to Ms. C.-Y. Chien of the Ministry of Science and Technology (National Taiwan University) for assistance with TEM experiments.

1. Chemicals and Reagents

Hyaluronic acid (HA,), $CuCl_2.2H_2O$ (98%), $RuCl_3.2H_2O$ (98%), NaOH (97%), H_2O_2 (30%), 3,3,5,5`-tetramethylbenzidine (TMB, 99%), $KMnO_4$ (98.5%), H_2SO_4 (99%), 2,7-dichlorofluorescein diacetate (DCFH-DA) were obtained from Sigma-Aldrich (St. Louis, U.S.A.). Apoptosis kit with annexin V-FITC and PI, DAPI, were purchased from Thermo Fisher Scientific (Waltham, MA, U.S.A.). Anhydrous acetone (99.8%), trichloro benzyl chloride (98%), and triethylamine (TEA, 99%) were obtained from 11 Technology (China). 3-mercaptopropionic acid (99%), trifluoroacetic acid (98%), 4-dimethyl aminopyridine (DMAP, 99%), N, N-Dicyclohexylcarbodiimide (DCC, 98%) were also obtained from 11 Technology (China). Dichloromethane (DCM, 99.5%), 30% H_2O_2 were obtained from Fluka. WST-1 (4-[3-(4-iodophenyl)-2H-5-tetrazolio]-1,3-benzenesulfonate assay was purchased from Roche Applied Science (Penzberg, Germany). Camptothecin (CPT, 97%), 3,3`,5,5`-tetramethylbenzidine dihydrochloride hydrate (TMB, 99%), glutathione (GSH, 98%), deuterium oxide (D_2O, 99.9%), 5,5`-dithiobis (2-nitrobenzoic acid) (DTNB, 98%), minimum essential medium Engle (MEME), high-glucose Dulbecco`s modified Eagle`s medium (DMEM, 90%), Dimethyl sulfoxide (DMSO, 99.8%), 1,4-diphenyl-2,3-benzofuran, (DPBF, 99.95%) were purchased from J.T. Baker, USA. The nucleic staining probes such as NucGreen^{TM}, and 4′,6-diamidino-2-phenylindole (DAPI, 98%) dead cell nucleic acid stain were purchased from ABP Biosciences, USA. 2,2,6,6-tetramethylpiperidine (TEMP, $\geq$99%), 2,7-dichlorofluorescein diacetate (DCFH-DA, >97%), tris(4,7-diphenyl-110-phenanthroline)ruthenium(II) dichloride ($Ru(dpp)_3Cl_2$), 5,5-Dimethyl-1-pyrroline N-oxide (DMPO, $\geq$97%), N, N-dimethylformamide (DMF, $\geq$99.8%) were ordered from Acros, USA. All other chemicals and reagents used were of analytical grade.

2. ROS-cleavable Camptothecin derivatives (CPT-TK-COOH)

Following the recently reported literature [1 2] a thioketal linker (TK) and ROS-responsive CPT-TK were synthesized. Briefly, 39.68 mmol of anhydrous 3-mercaptopropionic acid, 19.48 mmol of anhydrous acetone, and 11.68 mmol of trifluoroacetic acid were poured into a sample vial and gently stirred at room temperature for 1 h until crystallization occurred. The resulting TK was washed with hexane and cold water, filtered through a 0.45μM filter, freeze-dried, and stored in a refrigerator for future use.

To synthesize the ROS-sensitive CPT-TK, camptothecin was reacted with TK following modifications of previously reported methods [1 2], and Yamaguchi esterification [3] was used. Specifically, 0.5 mmol of TK powder was dissolved in 10 mL of DMF, followed by the addition of 1.5 mmol triethylamine, 0.5 mmol trichlorobenzoylchloride, and 0.1 mmol DMAP. The mixture was then mixed at room temperature for 10 min, and 0.25 mmol of the CPT solution (87.1 mg CPT, 10 mL DMF) was injected into the mixture and stirred at room temperature for 24 h. The reaction was neutralized with DI water before isolation using DCM and saturated brine water. Finally, excess water was removed through suction filtration with sodium sulfate, and the CPT-TK was concentrated using a rota-evaporator to form a solid and the crude product was stored in a refrigerator for characterization and subsequent use.

3. Detection of GSH at intracellular level

To validate intracellular GSH depletion properties of as-prepared material, we adopt our previously used protocol [4 5 6]. MDA-MB-231 cells were seeded at a density of 1×10^5 cells per well in 24 well culture plates with 2.0 mL of DMEM and then incubated for 24 h. The DMEM-grown cells were classified as the control group, and the original media was replaced with fresh DMEM that contained RCpCCPT in a range of concentrations (from 0 to 100 ppm). The cells were rinsed with PBS and then trypsin was used to separate them after a 12-h incubation period.

The cells were then centrifuged for 5 min. at 10,000 rpm after being suspended in 0.8 mL of PBS and chilled to -4 ºC. The cells were taken out of the supernatant and resuspended in 1 mL of PBS before being frozen and thawed three times to lyse them. Following that, 2 mL of the DTNB reagent (50 mM DTNB, the GSH assay kit) was added to 500 µL of the cell homogenates. The supernatant was gathered and subjected to a 10 min centrifugation at 10,000 rpm to test for GSH. 100 µL of the prepared sample and the reagent were added to each of the 96 test wells in the 96-well plate. A series of blank reagents were also added in parallel to the tested wells, along with a reagent for the control group. In the meantime, standard groups of 2 µL of DNTB reagent and 100 µL out of 500 µL of GSH standard solution (0, 20, 40, 60, 80, and 100 µM) were established. A Bio-Tek microplate absorption spectrometer was used to measure the optical density (OD) values of each well at 412 nm after well mixing and waiting 5 min. The standard calibration graph was produced by plotting a series of GSH-added concentrations versus OD. Equation [4] [5] illustrates the estimation of the proportion of GSH found in cells after prodrug administration.

$$\text{GSH content after treatment (\%)} = \frac{(\boldsymbol{OD}\ prodrug - \boldsymbol{OD}\ blank)}{(\boldsymbol{OD}\ control - \boldsymbol{OD}\ blank)} \times 100\%$$

4. Characterization methods

The absorption and emission properties of the prepared $Ru_xCu_{1-x}O_2$ NDs and RCpCCPT were studied using a steady state Ultraviolet-Visible spectrometer (Model: FP-6500; manufacturer: JASCO.CO.Ltd) and photoluminescence spectroscopy (Model: Fluorolog-3; manufacturer: HORIBA Jobin Yvon). The morphological study was carried out by transmission electron microscopy (TEM) and high-resolution TEM; model Tecnai F20G2 FETEM, Philips: model JEM-2100F, JEOL). X-ray Photoelectron Spectroscopy (XPS) was conducted using an ESCALAB 250 (VG Scientific), and X-ray diffraction (XRD) was performed using a Bruker D8 Discover diffractometer equipped with monochromatized Cu Ka radiation (wavelength

1.54 Å). The surface characterization of the obtained RCpCCPT was done using Fourier transform infrared (FT-IR) spectroscopy Nicolet 5700 FT-IR spectrometer (Thermo electron, USA). Nuclear magnetic resonance spectroscopy (^{1}H NMR) was conducted to confirm the conformation of TK and CPT-TK formation. The laser irradiated experiment was conducted using a 671 nm laser source (Led & Tec, MGL-III-532-100mW, Hi-Tech Optoelectronics Co., Ltd. Beijing, China).

(a)

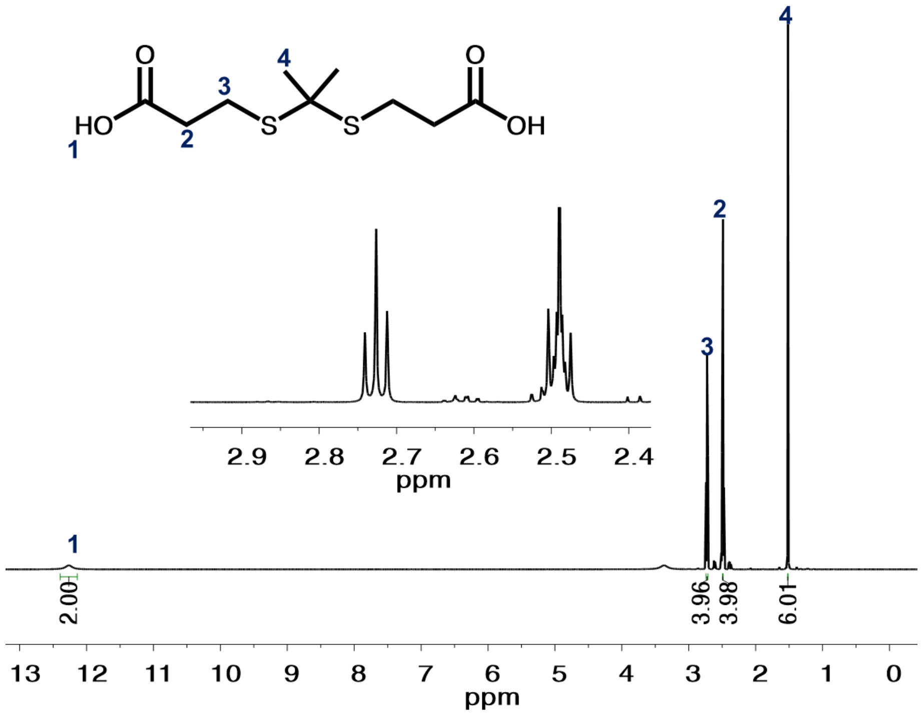

(b)

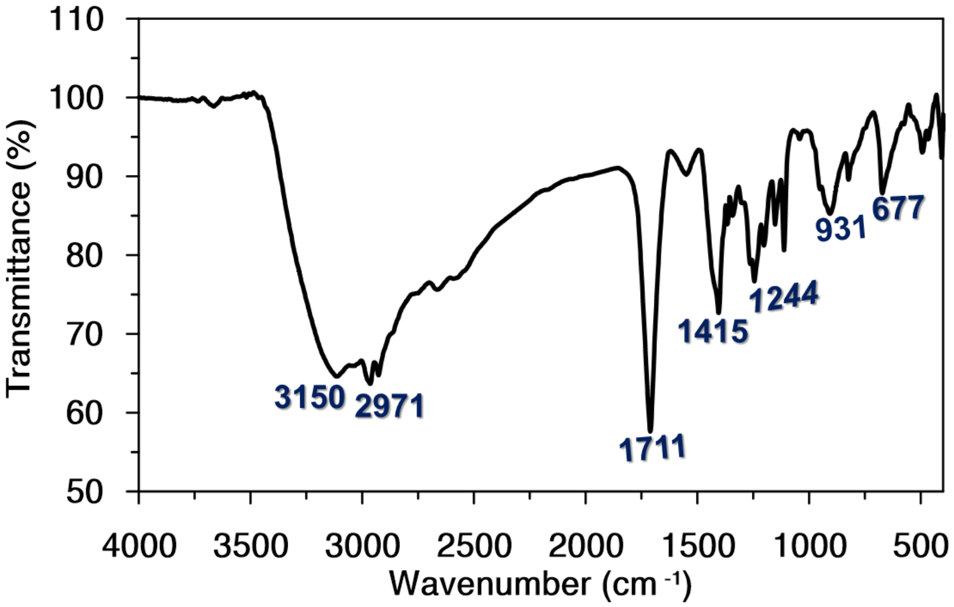

Figure S1. (a) ^{1}H NMR and (b) FT-IR spectra of TK.

^{1}H NMR spectra (400 MHz, DMSO-d_6) δ (ppm): 12.40 (broad, 1H), 2.74 (t, 4H), 2.49 (t, 4H), 1.50 (s, 6H), while FTIR spectra have confirmed the presence of COOH, CH_2, C=O, -CH_3, C-S, corresponding to broad peak centered 3250 (3450- 2600 cm^{-1}), 2968 cm^{-1}, 1707 cm^{-1}, 1407 and 673 cm^{-1} of produced TK (Figure S1).

(a)

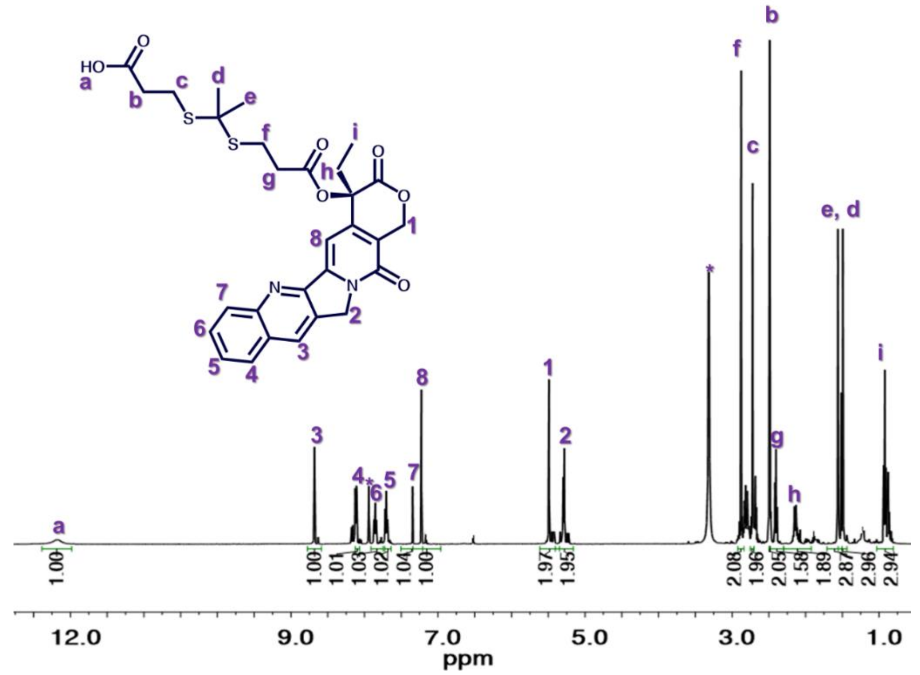

(b)

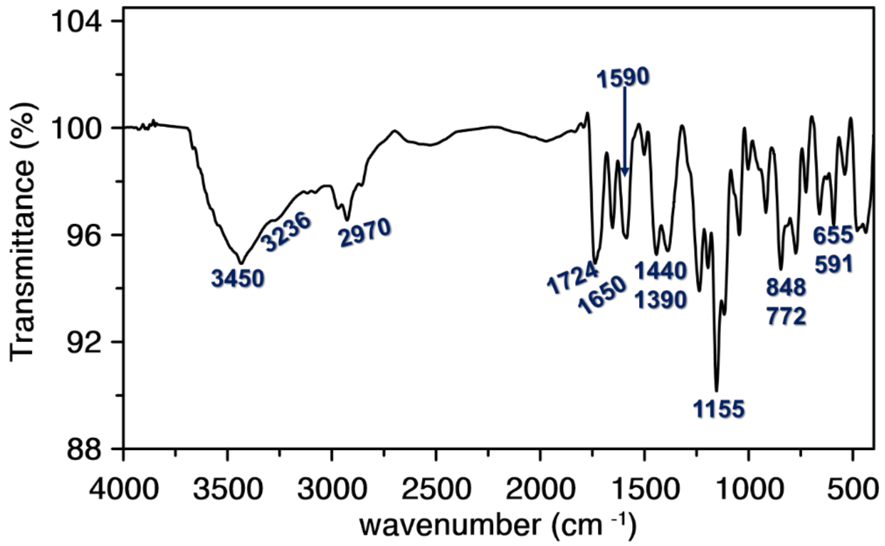

Figure S2. (a) ^{1}H NMR and (b) FT-IR spectra of CPT-TK.

^{1}H NMR (400 MHz, DMSO-d_6) δ (ppm): 12.41 (b, 1H), 8.82 (s, 1H), 8.50 (s, 1H), 8.10 (d, 1H), 7.92 (t, 1H), 7.88 (t, 1H), 7.40 (d, 1H), 6.90 (s, 1H), 5.41 (s, 2H), 5.11 (s, 2H), 2.89 (t, 2H), 2.72 (t, 2H), 2.49 (t, 2H), 2.41 (t, 2H), 2.15 (q, 2H), 1.56 – 1.49 (s, 6H), 0.94 (t, 3H) (Fig. S2). In the FTIR spectra (Figure S2), the presence of COOH stretch, aryl ring, and ester carbonyl confirmed the structure of the synthesized CPT-TK. The strong peak at 3433 cm^{-1}

indicated the presence of COOH stretch, while the peak at 3270 cm^{-1} and the overtone in the region of 2000 – 1800 cm^{-1} confirmed the presence of the aryl ring. The carbonyl peaks in the region of 1736 – 1585 cm^{-1} indicated the presence of carboxylic acid and ester carbonyl, thereby confirming the structure of synthesized CPT-TK

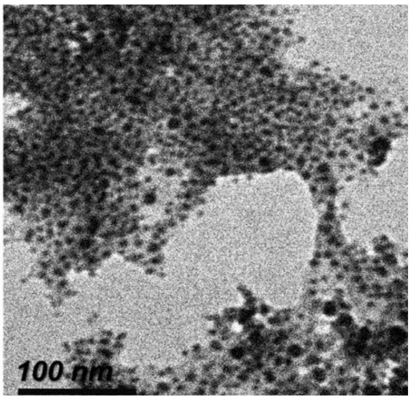

Figure S3. TEM image of RCp NDs

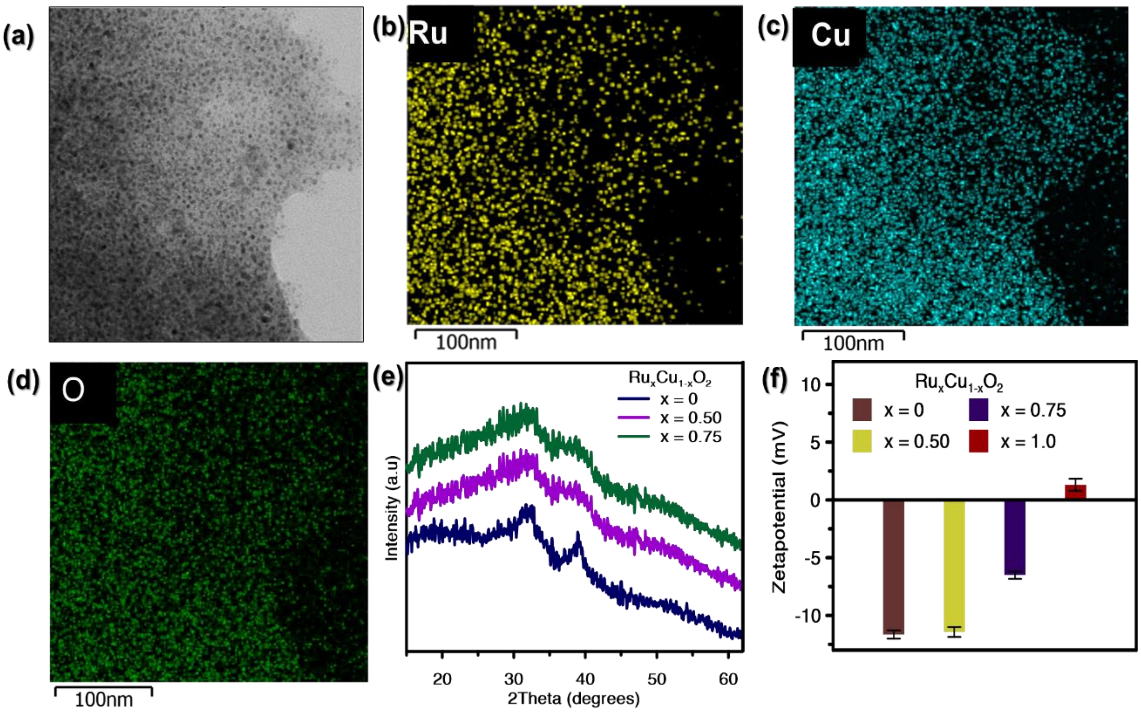

Figure S4. (a), HRTEM image and its elemental mapping images of (b) Ru, (c) Cu, and (d) O as described in RCp NDs. (e) XRD of $Ru_xCu_{1-x}O_2$ NDs (x = 0.0, 0.50, 0.75). (f), Zeta potential of $Ru_xCu_{1-x}O_2$ NDs (x = 0.0, 0.50, 0.75, 1.00).

Table S1. The Cu^{+} to Cu^{2+} and Ru^{2+} to Ru^{3+} ratios calculated from XPS data at selected performance ($Ru_xCu_{1-x}O_2$, x = 0.50) in RCp NDs

No.	Valence state or species	Binding energy	[a] Percentage of peak area for each species per metal	Mixed Oxidation state
1	Cu $2p_{3/2}$ (II)	933.5 eV	60.69%	Ratios of Cu^{+} to Cu^{2+} = 0.64
	Cu $2p_{1/2}$ (I)	952.0 eV	39.30%	
2	Ru $3p_{3/2}$ (II)	462.2 eV	40.21%	More dominant with Ru^{2+}/Ru^{3+} = 1.12
	Ru $3p_{1/2}$ (III)	484.3 eV	35.81%	
	Ru $3p_{3/2}$ (IV)	468.5 eV	14.9%	
	Ru (satellite peak)	458.1 eV	10.12%	

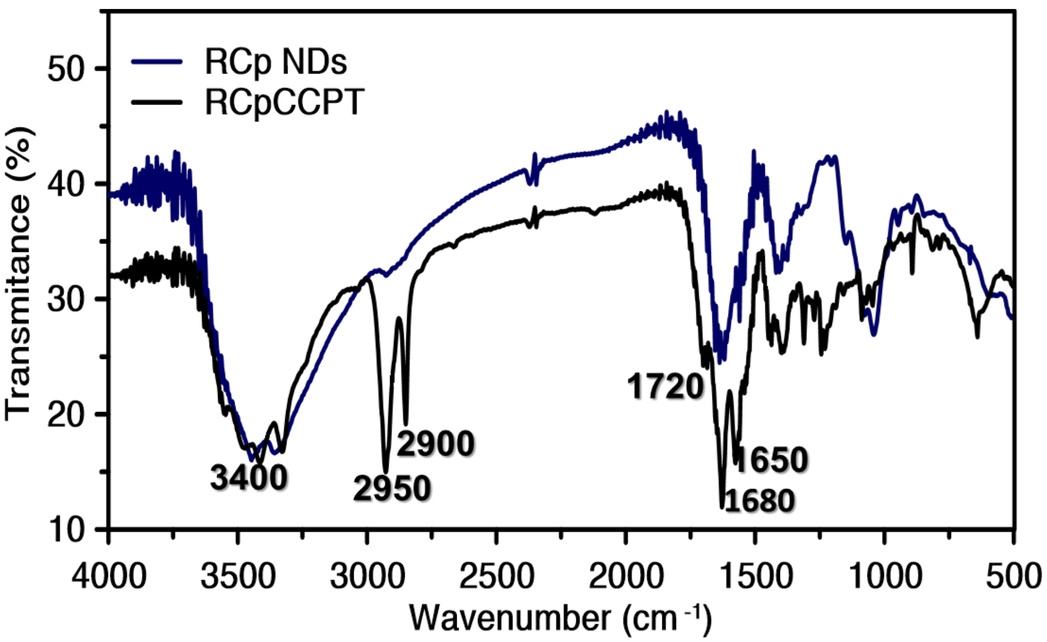

Figure S5. FT-IR spectra of RCp NDs and RCpCCPT.

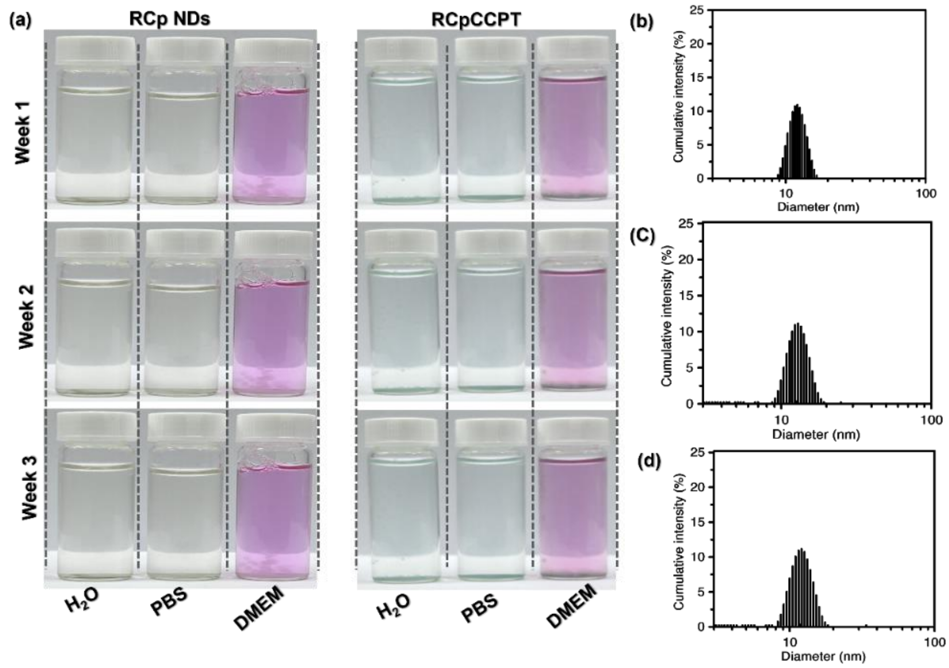

Figure S6. (a) RCp NDs and RCpCCPT stability in different solvents. DLS measurement of RCpCCPT; (b) in water, (c) in PBS, (d) in DMEM after two weeks.

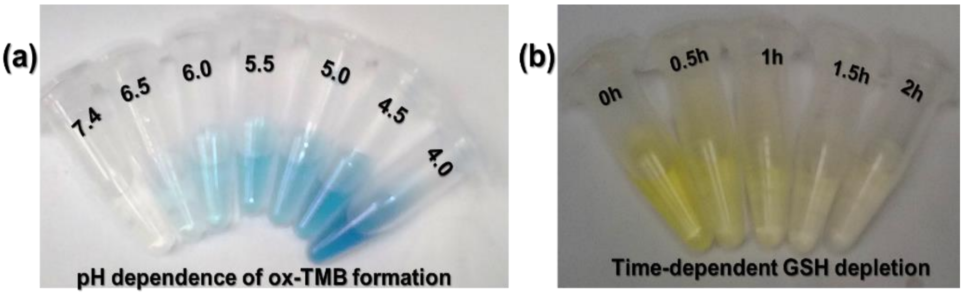

Figure S7. (a) Depending on the pH levels, TMB probe oxidation, and (b) GSH depletion as the incubation time of GSH + DTNB increases in the presence of RCp NDs.

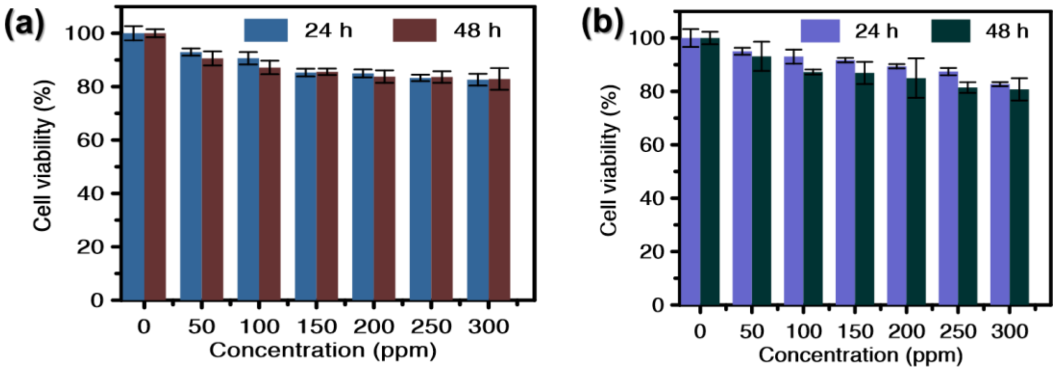

Figure S8. Cytocompatibility experiment Ru-Cu oxides prepared without adding H_2O_2 for intracellular studies as comparison test; (a) MDA-MB-231 cell lines, (b) 4T1 cell lines

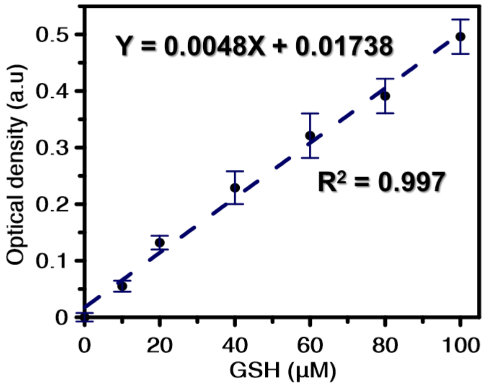

Figure S9. Intracellular quantification of GSH. The standard curve of optical density (OD) versus the different concentrations of GSH using a DTNB probe at 412 nm, (SD, n =3)

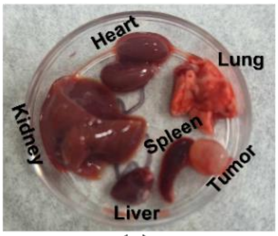

(a)

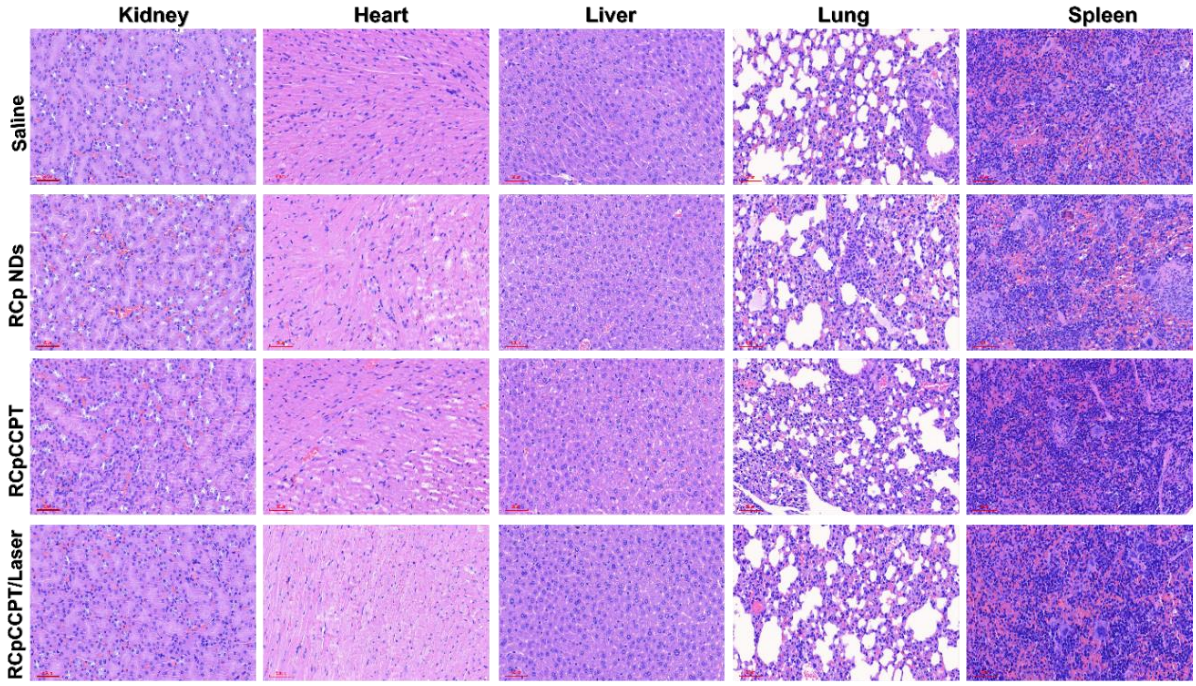

(b)

Figure S10. (a) representative photo of tumor-bearing mice main organs and tumor harvested on day 21 of treatments, (b) (b) H&E examination of the heart, lung, liver, spleen, and kidney excised from MDA-MB-231 tumor-bearing mice after 21 days treated with saline, RCp NDs, RCpCCPT, or RCpCCPT with laser after intravenous administration. Scale bar 60 μm

Table S2. Blood and biochemical data of serum analysis of the MDA-MB-231 tumor-bearing mice after 21 days treated with saline, RCp NDs, RCpCCPT, or RCpCCPT with laser intravenous administration.

Parameter	Saline	RCp NDs	RCpCCPT	RCpCCPT/Laser	Reference value
	Mean + SD	Mean + SD	Mean + SD	Mean + SD	
AST, U/L	102 + 24.97	75.00 + 9.85	111.75+38.1	87.66 + 6.02	59 - 247
ALT, U/L	34.33 + 4.72	32.00 + 1.00	32.75 + 2.87	38.33 + 2.30	28 - 132
BUN, mg/dL	25.66 + 2.30	24.00 + 1.73	27.00 + 3.00	23.66 + 1.15	18 - 29
CREA, mg/dL	0.20 + 0.00	0.133 + 0.06	0.30 + 0.11	0.17 + 0.06	0.2 - 0.8

Abbreviations: *AST, aspartate aminotransferase; ALT, alanine transaminase; BUN, blood urea nitrogen; CREA, creatinine +SD (n =3)*

References

(1) Yue, C.; Yang, Y.; Zhang, C.; Alfranca, G.; Cheng, S.; Ma, L.; Liu, Y.; Zhi, X.; Ni, J.; Jiang, W. ROS-Responsive Mitochondria-Targeting Blended Nanoparticles: Chemo-and Photodynamic Synergistic Therapy for Lung Cancer with on-Demand Drug Release Upon Irradiation with a Single Light Source. *Theranostics* **2016**, *6* (13), 2352.

(2) Dirersa, W. B.; Getachew, G.; Wibrianto, A.; Rasal, A. S.; Gurav, V. S.; Fahmi, M. Z.; Chang, J.-Y. Molybdenum-oxo-Sulfide Quantum Dot-Based Nanocarrier: Efficient Generation of Reactive Oxygen Species via Photo/Chemodynamic Therapy and Stimulus-Induced Drug Release. *J. Colloid Interface Sci.* **2023**, *647*, 528-545.

(3) Inanaga, J.; Hirata, K.; Saeki, H.; Katsuki, T.; Yamaguchi, M. A Rapid Esterification using Mixed Anhydride and Its Application to Large-Ring Lactonization. *Bul. Chem. Soc. Japan* **1979**, *52* (7), 1989-1993.

(4) Dirersa, W.; Getachew, G.; Hsiao, C.-H.; Wibrianto, A.; Rasal, A.; Huang, C.-C.; Chang, J.-Y. Surface-Engineered $CuFeS_2$/Camptothecin Nanoassembly with Enhanced Chemodynamic Therapy via GSH Depletion for Synergistic Photo/Chemotherapy of Cancer. *Mater. Today Chem.* **2022**, *26*, 101158.

(5) Shen, W.; Liu, W.; Yang, H.; Zhang, P.; Xiao, C.; Chen, X. A Glutathione-Responsive Sulfur Dioxide Polymer Prodrug as a Nanocarrier for Combating Drug-Resistance in Cancer Chemotherapy. *Biomater.* **2018**, *178*, 706-719.

(6) Xiong, Y.; Xiao, C.; Li, Z.; Yang, X. Engineering Nanomedicine for Glutathione Depletion-Augmented Cancer Therapy. *Chem. Soc. Rev.* **2021**, *50* (10), 6013-6041.
